# Supplementary material for: Polymorphic Region-Specific Antibody for Evaluation of Affinity-Associated Profile of Chimeric Antigen Receptor
Source: Mol Ther Oncolytics. 2020 Apr 14;17:293–305. doi: 10.1016/j.omto.2020.04.004 (PMC7191539; doi:10.1016/j.omto.2020.04.004)
Supplement: Document S1. Figures S1–S3 [file mmc1.pdf]

OMTO, Volume *17*

Supplemental Information

Polymorphic Region-Specific Antibody for Evaluation of Affinity-Associated Profile of Chimeric Antigen Receptor

Chungyong Han, Beom K. Choi, Seon-Hee Kim, Su-Jung Sim, Seongeun Han, Bomi Park, Yohei Tsuchiya, Masaki Takahashi, Young H. Kim, Hyeon-Seok Eom, Tetsuya Kitaguchi, Hiroshi Ueda, and Byoung S. Kwon

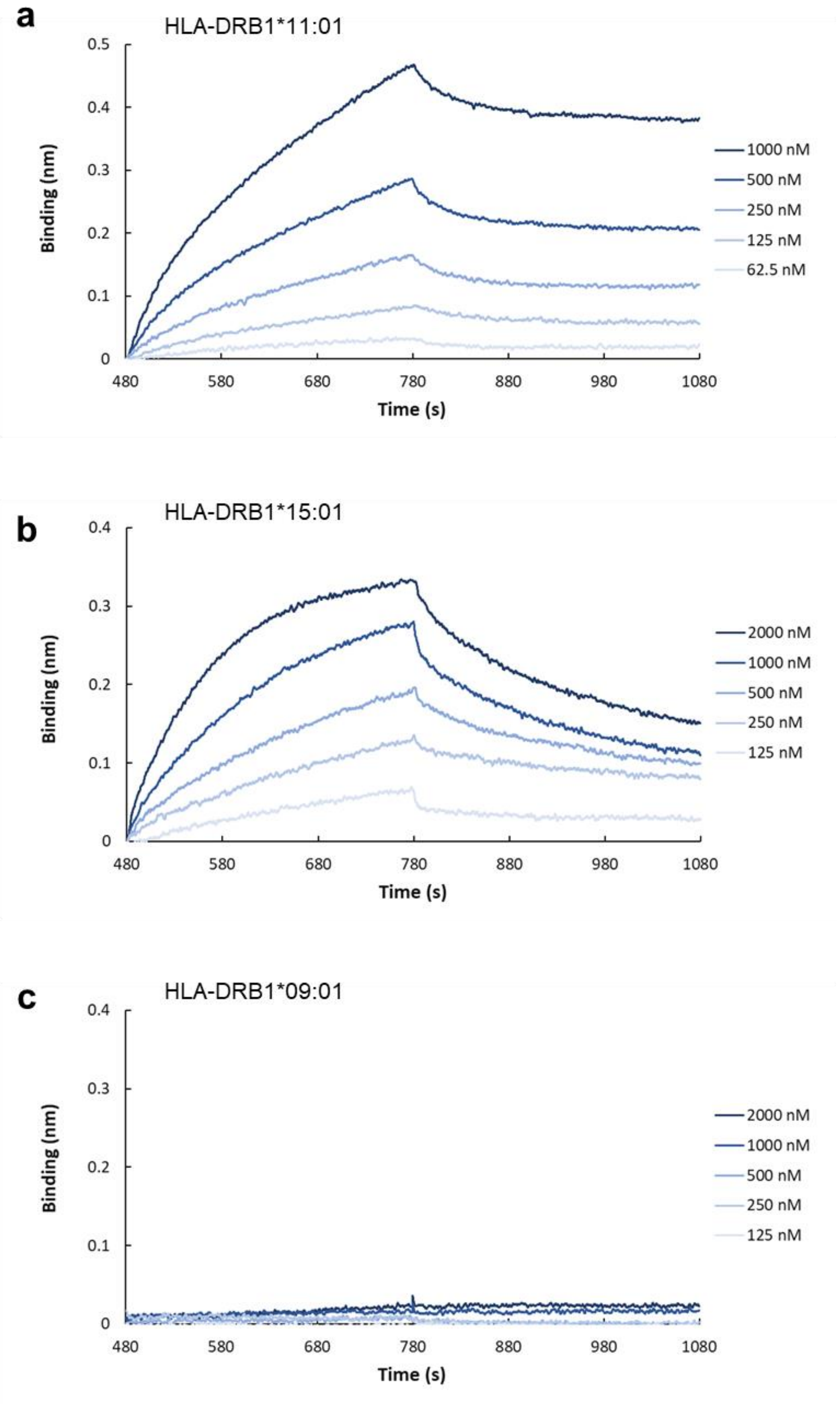

Supplementary Figure 1. The result of biolayer interferometry analysis. Protein interaction between immobilized HLA-DR (HLA-DRB1*11:01 [**a**], HLA-DRB1*15:01 [**b**] or HLA-DRB1*09:01 [**c**] complexed with HLA-DRA*01:01 and CLIP) and MVR-scFv was measured.

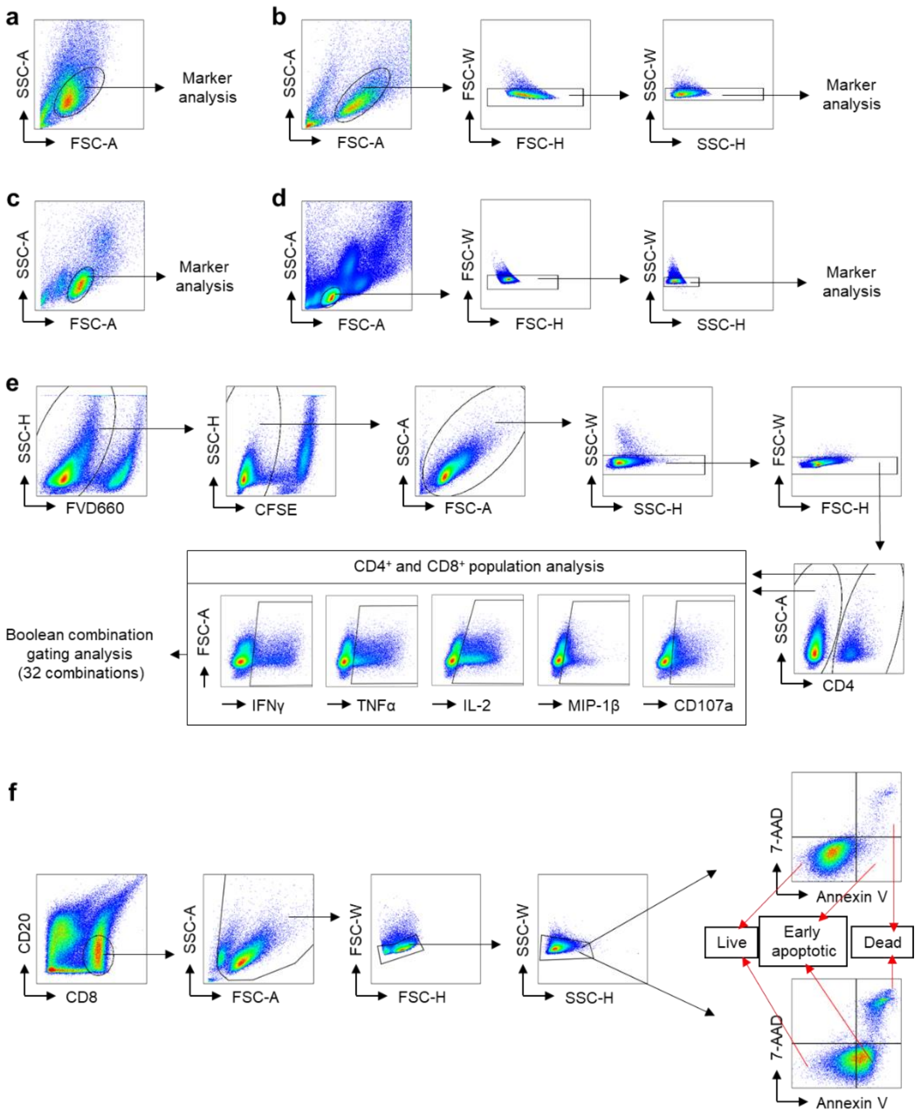

Supplementary Figure 2. Gating strategy. (a) To analyze cell lines using FACSCalibur (**Figure 1b,c and 3a**), viable cell population was gated and analyzed. **(b)** To analyze cell lines using FACSVerse (**Figure 4b and 5a,b**), singlet population in viable cells was gated and analyzed. **(c)** To analyze peripheral blood mononuclear cells using FACSCalibur (**Figure 3b**), viable cell population was gated and analyzed. **(d)** To analyze T cells using FACSVerse (**Figure 5c**), singlet population in viable cells was gated and analyzed. **(e)** Boolean combination gating for the polyfunctionality analysis (**Figure 6a,b**). Viable cell population (fixable viability dye eFluor 660-negative; FVD660$^-$) was gated and further separated from carboxyfluorescein succinimidyl ester (CFSE)-labeled LCLs. Within singlet population, CD4$^+$ or CD4$^-$ (i.e. CD8$^+$) cells were analyzed for each marker expression. The frequency of cell population with multiple marker expression was calculated by Boolean combination gating analysis. **(f)** Gating for the evaluation of apoptotic/dead cell frequency (**Figure 6g,h**). CD8$^+$ CAR-T population was gated by excluding CD20^{high} LCLs. FSC-A/SSC-A gating was performed to exclude debris. Following singlet gating (FSC-H/FSC-W and SSC-H/SSC-W), the frequency of live (Annexin V$^-$/7-AAD$^-$), early apoptotic (Annexin V$^+$/7-AAD$^-$), and dead (Annexin V$^+$/7-AAD$^+$) cells among population was determined.

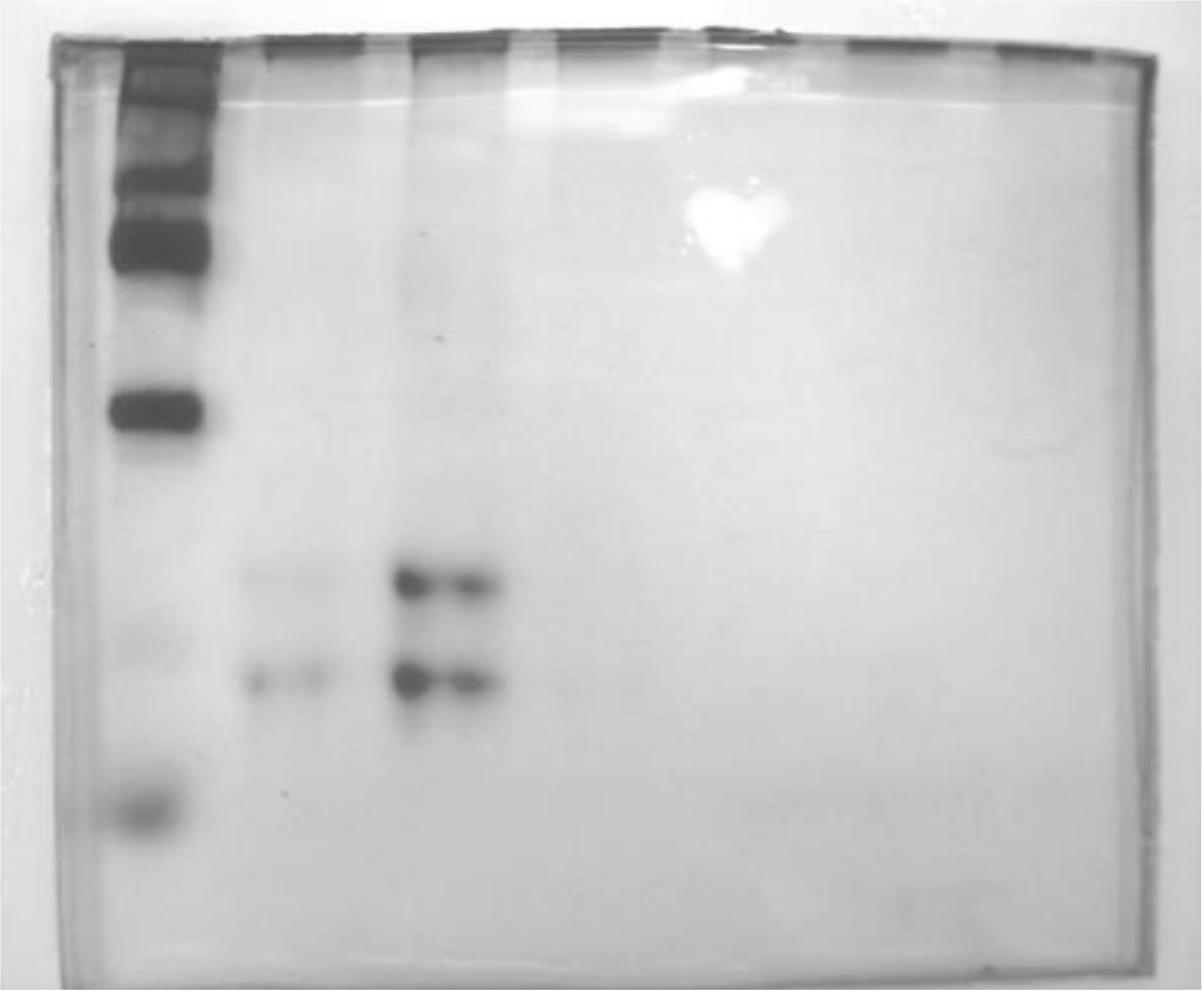

Supplementary Figure 3. Original untrimmed image of Figure 2b
