# Supplementary material for: Polymorphic Region-Specific Antibody for Evaluation of Affinity-Associated Profile of Chimeric Antigen Receptor
Source: Mol Ther Oncolytics. 2020 Apr 14;17:293–305. doi: 10.1016/j.omto.2020.04.004 (PMC7191539; doi:10.1016/j.omto.2020.04.004)
Supplement: Document S2. Article plus Supplemental Information [file mmc2.pdf]

Molecular Therapy
Oncolytics
Original Article

Polymorphic Region-Specific Antibody for Evaluation of Affinity-Associated Profile of Chimeric Antigen Receptor

Chungyong Han,[1,9] Beom K. Choi,[2,9] Seon-Hee Kim,[1,9] Su-Jung Sim,[1] Seongeun Han,[1] Bomi Park,[2] Yohei Tsuchiya,[3] Masaki Takahashi,[4] Young H. Kim,[2,5] Hyeon-Seok Eom,[6] Tetsuya Kitaguchi,[7] Hiroshi Ueda,[7] and Byoung S. Kwon[1,5,8]

[1]Division of Tumor Immunology, Research Institute, National Cancer Center, Goyang, Republic of Korea; [2]Biomedicine Production Branch, Research Institute, National Cancer Center, Goyang, Republic of Korea; [3]Interdisciplinary Graduate School of Science and Engineering, Tokyo Institute of Technology, Yokohama, Japan; [4]Graduate School of Life Science and Technology, Tokyo Institute of Technology, Yokohama, Japan; [5]Eutilex Institute for Biomedical Research, Eutilex, Seoul, Republic of Korea; [6]Center for Hematologic Malignancy, Hospital, National Cancer Center, Goyang, Republic of Korea; [7]Laboratory for Chemistry and Life Science, Institute of Innovative Research, Tokyo Institute of Technology, Yokohama, Japan; [8]Department of Medicine, Tulane University Health Sciences Center, New Orleans, LA, USA

Antibody applications in cancer immunotherapy involve diverse strategies, some of which redirect T cell-mediated immunity via engineered antibodies. Affinity is a trait that is crucial for these strategies, as optimal affinity reduces unwanted side effects while retaining therapeutic function. Antibody-antigen pairs possessing a broad affinity range are required to define optimal affinity and to investigate the affinity-associated functional profiles of T cell-engaging strategies such as bispecific antibodies and chimeric antigen receptor-engineered T cells. Here, we demonstrate the unique binding characteristic of the developed antibody clone MVR, which exhibits robust binding to B-lymphoid cell lines. Intriguingly, MVR specifically recognizes the highly polymorphic human leukocyte antigen (HLA)-DR complex and exhibits varying affinities that are dependent upon the *HLA-DRB1* allele type. Remarkably, MVR binds to the conformational epitope that consists of two hypervariable regions. As an application of MVR, we demonstrate an MVR-engineered chimeric antigen receptor (CAR) that elicits affinity-dependent function in response to a panel of target cell lines that express different *HLA-DRB1* alleles. This tool evaluates the effect of affinity on cytotoxic killing, polyfunctionality, and activation-induced cell death of CAR-engineered T cells. Collectively, MVR exhibits huge potential for the evaluation of the affinity-associated profile of T cells that are redirected by engineered antibodies.

INTRODUCTION

Monoclonal antibodies are useful agents for various applications. One such application is cancer immunotherapy, which utilizes an antibody in its intact form to activate or block a targeted receptor or in an engineered form to engage T cells and elicit anti-tumor immunity (e.g., bispecific antibody, bispecific T cell engager and chimeric antigen receptor [CAR]).[1–3]

Antibodies generally bind to target antigens with high affinity (K_D in the nM range) to elicit target-specific activities. In the case of T cell-engaging strategies, however, this high affinity is often accompanied by serious on-target off-tumor side effects, since these strategies redirect cytotoxic T cells that mediate massive immune responses.[4–6] Recent studies have suggested that on-target off-tumor side effects can be reduced by adjusting the affinity of bispecific antibodies and CARs.[7–10] However, an optimal affinity range, in which the T cells mediate maximal therapeutic effects while minimizing side effects, has not been fully investigated because of the lack of antibody-antigen pairs with a broad affinity range.

In this study, we describe the characteristics of a newly developed antibody clone, MVR, which specifically binds to the HLA-DR molecule. Since MVR recognizes a conformational epitope located in the polymorphic region, this antibody demonstrates a broad spectrum of affinity to different alleles of the β-chain of HLA-DR (DRβ). We also demonstrate an application of MVR, in which the correlation between affinity and the function of 4-1BB-containing second-generation CAR-engineered T cell (CAR-T) is evaluated. This HLA-DR-specific antibody can be used to study the affinity-related functional profiles of T cell-engaging strategies.

RESULTS

A Newly Developed Antibody Clone, MVR, Selectively Binds to B-Lymphoid Cells

We developed antibodies specific to the B-lymphoid lineage by immunizing BALB/c mice with human-derived B-lymphoma, L3055.[11] We fused the splenocytes of mice with SP2/0 myeloma cells, thereby generating hybridomas to screen the specificity of the

Received 26 March 2020; accepted 7 April 2020;
https://doi.org/10.1016/j.omto.2020.04.004.

[9]These authors contributed equally to this work.

Correspondence: Byoung S. Kwon, Eutilex Institute for Biomedical Research, Eutilex, Seoul, Republic of Korea.
E-mail: bskwon@eutilex.com

This is an open access article under the CC BY license (http://creativecommons.org/licenses/by/4.0/).

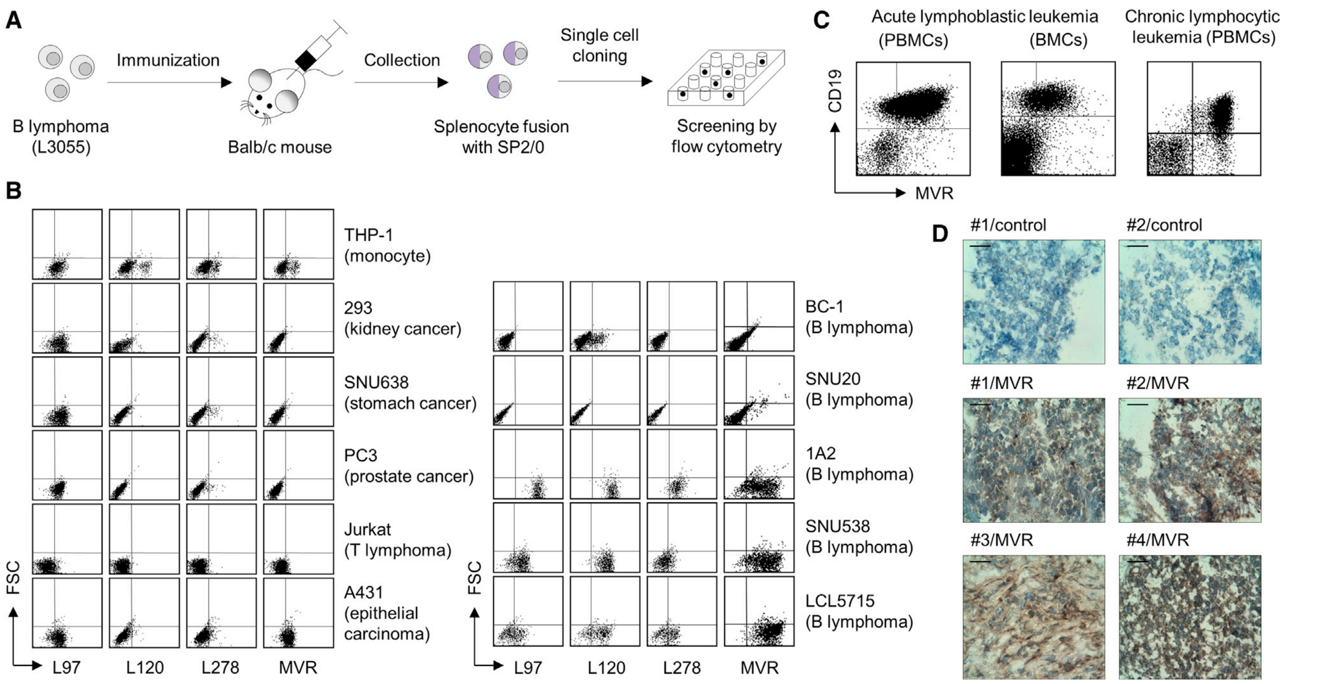

Figure 1. The Antibody Clone, MVR, Robustly Binds to B-Lymphoid Cells

Antibody clones derived from B lymphoma-immunized mice were evaluated for their binding capacity to various cell lines and tissues. (A) A schematic for antibody development. BALB/c mice were immunized with a B lymphoma cell line, L3055. Splenocytes collected from the mice were fused with SP2/0 cells, and single cell was cloned for antibody screening. The screening isolated four antibody clones (L97, L120, L278, and MVR) based on their binding to L3055. (B) Evaluation of antibody binding to various cell lines. The indicated cell lines were stained with L97, L120, L278, or MVR and analyzed by flow cytometry. (C) Evaluation of MVR binding to leukemic cells. Patient-derived PBMCs and BMCs were co-stained with anti-CD19 and MVR and analyzed by flow cytometry. (D) The evaluation of MVR binding on lymphoma tissues. Tumor tissues derived from diffuse large B cell lymphoma patients were stained with diaminobenzidine using MVR and HRP-conjugated anti-mouse IgG antibody and counterstained with hematoxylin. Tissues from four patients (indicated by number) were used. Control indicates staining without MVR. Scale bar, 50 μm.

antibodies (Figure 1A). Four hybridoma clones (L97, L120, L278, and MVR) indicated selective binding to B-lymphomas compared to cell lines derived from various other tissues (Figure 1B). Notably, MVR showed the highest binding to 3 out of 5 B-lymphoma cell lines (1A2, SNU538, and LCL5715). We further examined the specificity of MVR using patient-derived primary tissues. MVR showed robust binding to CD19-positive acute/chronic B cell leukemia (ALL/CLL) cells and to diffuse large B cell lymphoma (DLBCL) cells (Figures 1C and 1D). These results indicate that MVR specifically recognizes B-lymphoid cells.

MVR Recognizes HLA-DR on the Cell Surface

We sought to identify the target antigen bound by MVR using a proteomics approach (Figure 2A). MVR-binding proteins were affinity-purified from the lysate of LCL5715 using MVR-linked resin, and the eluate was analyzed using SDS-PAGE. The eluate separated into two bands ~30 and ~40 kDa in size (Figure 2B). Mass spectrometry using quadrupole time-of-flight (Q-TOF ESI-MS/MS) revealed that the two bands represented DRβ (band 1) and a mixture of the α chain of HLA-DR (DRα) and CD74 (band 2), respectively (Figure 2C).

CD74 is expressed as a homotrimer or a heterotrimer along with DRα and DRβ to form HLA-DR, the classical major histocompatibility complex II molecule.[12] To determine whether MVR genuinely recognizes HLA-DR, we co-stained LCL5715 with commercial HLA-DR-specific antibody and MVR. Confocal microscopy revealed a co-staining pattern of HLA-DR-specific antibody with MVR (Figure 2D), indicating that HLA-DR is the binding target of MVR.

MVR Shows a Broad Spectrum of Binding Strength Depending on the *HLA-DRB1* Allele

HLA-DR is a highly polymorphic protein complex that has diverse variants (2 DRα and 2,043 DRβ chains).[13] Owing to the hypervariability of DRβ, differences in the *HLA-DRB1* allele may result in various MVR binding affinities. Staining several HLA-DR-expressing cell lines (LCL5715, 1A2, and JVM-2) with a commercial HLA-DR antibody and MVR revealed the variation in MVR binding (Figure 3A), implying that MVR recognizes variable regions of HLA-DR. HeLa-CIITA (HeLa cells expressing class II major histocompatibility complex transactivator) bound more strongly to commercial HLA-DR antibody than to MVR, whereas LCL5715 and JVM-2 bound similarly to both antibodies. Of note, the expression level of the HLA-DR–class II-associated invariant chain peptide (CLIP) complex had no effect on MVR binding, suggesting that the peptide loaded onto HLA-DR does not alter MVR binding. We further investigated MVR binding to B cells with various *HLA-DRB1* alleles.

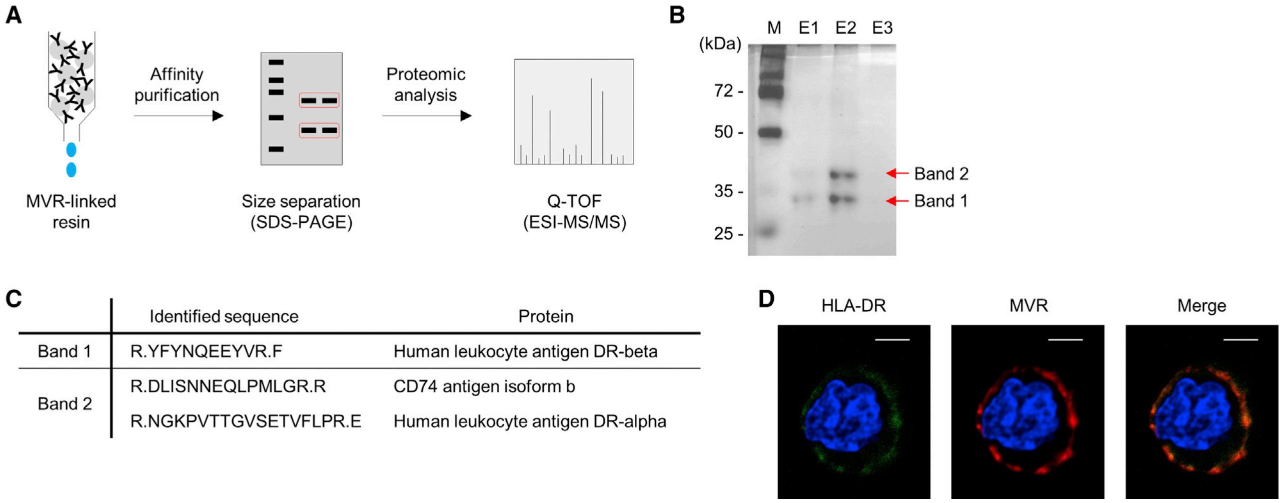

Figure 2. MVR Specifically Binds to the HLA-DR Complex

The protein specific to MVR was identified by mass spectrometry and evaluated for MVR binding. (A) A schematic for the identification of the MVR-binding protein. Affinity-purified LCL5715 lysate was size-separated and subjected to mass spectrometry for the identification of the MVR-binding protein. (B) The size-separation image of the MVR-binding protein. The first three elutes (E1-E3) collected from the affinity purification were separated by electrophoresis. (C) Proteins identified by mass spectrometry. Two bands in (B) were individually subjected to analysis. Full stop following Arg (R) indicate sites that were cleaved by trypsin digestion. (D) Images of LCL5715 cells stained with fluorochrome-labeled antibodies. LCL5715 cells were co-stained with anti-HLA-DR and MVR and analyzed by confocal microscopy. Scale bar, 5 μm.

Remarkably, peripheral blood-derived mononuclear cells (PBMCs) from healthy donors with different *HLA-DRB1* alleles showed a broad spectrum of binding strength (Figure 3B). Genotype analysis of these PBMCs identified *HLA-DRB1* alleles with strong or weak binding to MVR (Table 1). Of these types, DRB1*11:01 (an MVR strong binder), DRB1*15:01 (an MVR intermediate binder), and DRB1*09:01 (an MVR weak binder) were evaluated for binding strength via protein level by ELISA. The results revealed a stark contrast between the binding affinities of these three alleles (Figure 3C), supporting the idea that MVR recognizes the variable region of DRβ.

MVR Binds to a Polymorphic Region of HLA-DR

Next, we sought to identify the epitope recognized by MVR. We checked highly polymorphic regions in which a large number of amino acid variations exist among different *HLA-DRB1* alleles. In the 266-amino-acid-long sequence, region 1 (amino acids 38–45) and region 2 (amino acids 54–62) showed high variability among HLA-DRB1 types (Figure 4A). To verify whether both regions have an effect on MVR binding, we designed HLA-DRB1 chimera proteins comprised of fragments from two different types of HLA-DRB1 (Figure 4B). The designed chimera proteins consisted of the C-terminal of HLA-DRB1*11:01 and the N-terminal of HLA-DRB1*09:01, spanning either region 1 (09R1-11 chimera) or region 2 (09R1R2-11 chimera). We used the HLA-DRA-expressing dDR-CIITA cell line to evaluate the effect of HLA-DRB1 variation on MVR binding. The expression of the chimeras in dDR-CIITA revealed that both regions 1 and 2 affect MVR binding, implying that MVR recognizes a conformational epitope. Referencing the HLA-DR structure previously reported by Gunther et al.,[14] we found that the two regions comprise part of a β sheet structure inside the peptide-binding pocket of HLA-DR (Figure 4C). The sequence alignment of HLA-DRB1 amino acids, which were defined as strong or weak MVR-binders, indicated a characteristic feature within these regions (Figure 4D). Biolayer interferometry analysis estimated the extent of interactions between MVR and three HLA-DRB1 types (Table 2; Figure S1). K_D values for strong and intermediate MVR-binders were 88.1 nM ± 0.8 nM (HLA-DRB1*11:01) and 359 nM ± 4 nM (HLA-DRB1*15:01). The binding affinity of the weakest MVR-binder (HLA-DRB1*09:01) was below the detection limit of the system (>1 mM) and hence the K_D value for HLA-DRB1*09:01 was not determined. Collectively, these data suggest that MVR binds to a conformational epitope located in a highly polymorphic region on the HLA-DR complex.

MVR Enables the Evaluation of Affinity-Associated CAR-T Function

Antibody-antigen pairs with a broad affinity range enable the evaluation of the affinity-associated functions of T cell-engaging immunotherapeutic strategies, such as CAR-T. For this application, we generated MVR-engineered CAR-Ts and a list of target cells with various affinities. We generated a panel of lymphoblastoid cell lines (LCLs) using Epstein-Barr virus (EBV) transformation of PBMC-derived B cells with various MVR-binding strengths (Figure 5A). The generated LCLs yielded SD_{50} values from 0.1247 to 23.27 μM (Figure 5B), suggesting an affinity range with a wide distribution.[15] To prepare effector cells, we generated MVR CAR-transduced CAR-T (MVR CAR-T) using a 4-1BB-containing second-generation CAR construct.[9] Non-transduced T cells (NT-T; negative control) and CD19 CAR-transduced CAR-Ts (CD19 CAR-T; positive control) were generated and used as controls

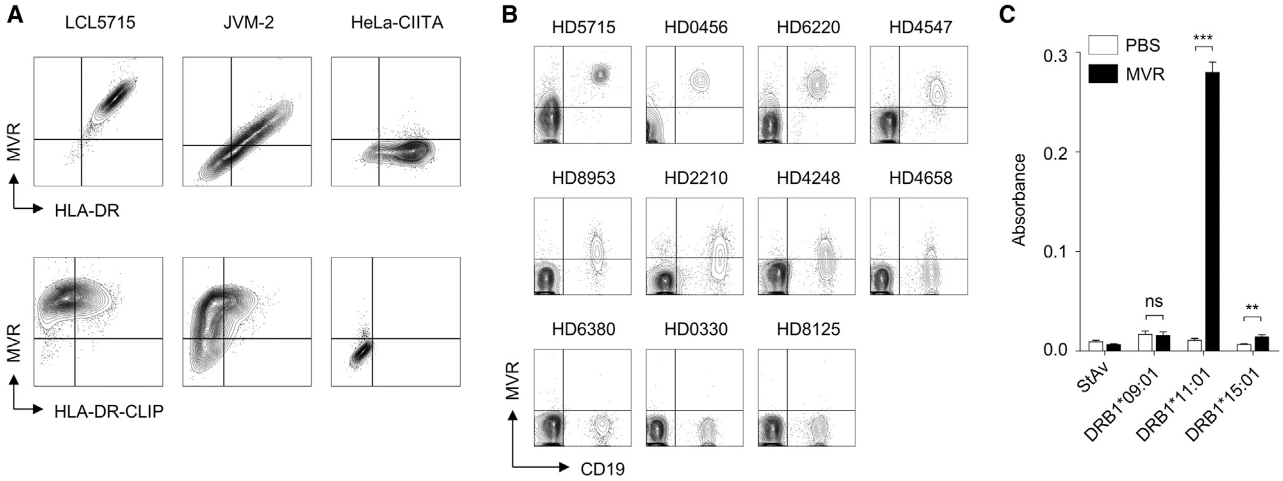

Figure 3. *HLA-DRB1* Alleles Affect the Binding Affinity of MVR

HLA-DR complexes with varying *HLA-DRB1* alleles were evaluated for MVR binding. (A) HLA-DR-expressing cell lines (LCL5715, JVM-2, and HeLa-CIITA) were co-stained with MVR and anti-HLA-DR or anti-HLA-DR-CLIP and analyzed by flow cytometry. (B) PBMCs from healthy volunteers with diverse *HLA-DRB1* alleles (Table 1) were co-stained with anti-CD19 and MVR and analyzed by flow cytometry. (C) MVR-target binding measured by ELISA. PBS or MVR was applied to the wells containing HLA-DR complexes with either HLA-DRA*01:01–HLA-DRB1*09:01–CLIP, HLA-DRA*01:01–HLA-DRB1*15:01–CLIP, or HLA-DRA*01:01–HLA-DRB1*11:01–CLIP. StAv, the negative control. n = 3 experimental replicates. Two-tailed unpaired Student's t test. ns, not significant; ***$p < 0.001$. Error bars indicate means ± SD.

(Figure 5C). For non-biased functional evaluation, the generated CAR-positive T cells were sorted by magnetic separation. Using these CAR-Ts and LCL pairs, we evaluated the killing efficiency of MVR CAR-Ts against a panel of LCLs with varying affinities. The killing efficiency increased depending on the effector to target (E:T) cell ratio in all LCLs (Figure 5D). Because CD19 CAR has no affinity variation among CD19 molecules in all LCLs, we could evaluate the affinity-related functional change by comparing the killing efficiency of CD19 CAR-Ts and MVR CAR-Ts. Of note, the killing activity of MVR CAR-Ts increased with MVR affinity and became saturated at a low SD_{50}, i.e., a high affinity range (Figure 5E), which is consistent with other CAR-T studies.[16–18]

CAR Affinity Correlates with CAR-T Polyfunctionality Below the Functional Plateau

Diverse CAR-target affinity is achieved by creating diversity in CAR constructs or target antigens. Either strategy results in undesirable variations among CAR-Ts or target cells. As MVR CAR-T system uses a single CAR construct, it minimizes inter-CAR-T variations and is suitable for investigating the qualitative change of CAR-T. Therefore, we evaluated the polyfunctionality change of CAR-T when stimulated with diverse affinities. The frequency of stimulated CAR-Ts expressing multiple functional markers (such as interferon-γ [IFN-γ], tumor necrosis factor alpha [TNF-α], interleukin-2 [IL-2], MIP-1β, and CD107a) was analyzed (Figures 6A and 6B). Overall, $CD4^+$ CAR-Ts showed higher polyfunctionality (the frequency of polyfunctional cells expressing more than four markers) than $CD8^+$ CAR-Ts (Figures 6C and 6D). CD19 CAR-T, which has the same binding affinity to six LCLs, indicated similarly distributed polyfunctionality, whereas MVR CAR-T increased the polyfunctionality at low to intermediate affinity and was saturated at a higher range as seen in the killing efficiency (Figure 5E). Notably, there was a polyfunctionality gap between CD19 and MVR CAR-Ts even at the saturating affinity, implying the existence of functional variations among CAR constructs and/or target antigens (i.e., CD19 versus HLA-DR; Figures 6C and 6D). We also observed an identical pattern of polyfunctionality fluctuation between CD19 and MVR CAR-Ts stimulated with LCLs of saturating affinity (LCL4658, LCL2210, LCL4248, and LCL5715), indicating target cell-intrinsic stimulation bias among LCLs. To eliminate these variations/biases and compare the sole affinity-associated change in polyfunctionality, we subtracted the polyfunctionality of CD19 CAR-T from that of MVR CAR-T for each LCL. A clear increase and plateau of polyfunctionality was evident following affinity escalation (Figures 6E and 6F). These data suggest that MVR can be applied for the evaluation of the affinity-associated functions of CAR-Ts.

CAR Affinity Correlates with the Rate of Activation-Induced Cell Death

Activation-induced cell death (AICD) impairs the *in vivo* persistence of CAR-T,[19–21] which is a key attribute for effective CAR-T therapy. To investigate the effect of CAR affinity on AICD, we stimulated MVR CAR-T with each LCL, and evaluated Annexin V binding and 7-AAD incorporation. CD19 CAR-T was also stimulated and evaluated as an affinity-fixed control. The proportion of dead cells (Annexin V^+/7-AAD^+) was similar between CD19 and MVR CAR-T regardless of the stimulating LCLs (Figures 6G and 6H). The apoptotic portion (Annexin V^+/7-AAD^-) of LCL-stimulated CD19 CAR-T did not significantly vary among LCLs and accounted for approximately 20% of the total CAR-T (Figure 6G). In contrast, the frequency of LCL-stimulated MVR CAR-T increased with CAR affinity from 18.83% to 51.63% (Figure 6H). This trend was retained

Table 1. *HLA-DRB1* Alleles from Cell Lines and Donors

Group Name	Donor ID	*HLA-DRB1* Allele Type	
Strong binders	LCL5715	080302[a]	150101
	HD5778	030101	110101[a]
	HD0456	100101[b]	110101[a]
	HD6220	090102[b]	110101[a]
	HD4547	080302[a]	090102[b]
Intermediate binders	HD8953	030101	120201
	HD2210	13	13
	HD4248	040501	150101
	HD4658	0908[c]	150101
Weak binders	HD6380	07[b]	14[b]
	HD0330	09[b]	14[b]
	HD8125	090102[b]	100101[b]
	HeLa-CIITA	010201[b]	010201[b]

[a]Alleles only observed among MVR strong binders.
[b]Alleles that exist among MVR weak binders.
[c]An allele that expresses null protein.

after the subtraction of the apoptotic frequency of CD19 CAR-T from that of MVR CAR-T (Figure 6I), implying that the AICD increase is unrelated to target cell-intrinsic stimulation bias among LCLs and is instead an affinity-driven result.

DISCUSSION

In 2018, we reported the first study on MVR that described intriguing characteristics of MVR CAR-T.[9] The study investigated the consequence of MVR CAR (which targets HLA-DR) transduction in the HLA-DR-expressing T cells. Without any knowledge on the binding epitope, we found that weak affinity CAR-T was desensitized through an "autotuning" process and could sense the antigen levels on the target cell surface. In the current study, we focused on describing the detailed characteristics of MVR clone and the affinity-associated profile of CAR-T. The developed antibody clone, MVR, bound to HLA-DR and specifically recognized cells of the B-lymphoid lineage. We found that MVR binding to the HLA-DR complex was affected by amino acid alterations at two highly polymorphic regions (amino acids 38–45 and 54–62) of DRβ. Since the binding was affected by two distant amino acid regions, leading to a wide affinity range among *HLA-DRB1* alleles, we hypothesized that MVR recognized a conformational epitope on HLA-DR. By analyzing amino acid variations within the epitope region, we chose B cells with different *HLA-DRB1* alleles and established a panel of LCLs that bound to MVR with various SD_{50} values, which is an index of the affinity of protein-protein interactions, ranging from 0.1247 μM to 23.27 μM depending on the *HLA-DRB1* allele.[15] Lastly, we used various MVR-LCL pairs to verify an affinity-associated functional profile of CAR-Ts.

HLA-DR, a classical major histocompatibility complex II molecule, is expressed strongly on the antigen-presenting cells, e.g., the dendritic cells, monocytes, and B cells. Owing to their expression profiles, HLA-DR-targeting monoclonal antibodies have been developed and tested via clinical studies of B cell leukemia and lymphoma.[22,23] Considering the application potential of HLA-DR antibodies in B cell malignancies, future evaluation of MVR in a therapeutic setting may yield insights into the optimal affinity of HLA-DR antibodies for clinical use; however, the broad HLA-DR expression profiles in diverse normal tissues must be carefully considered when targeting HLA-DR for therapeutic purposes.

The relationship between the CAR-T function and CAR-target affinity has been investigated previously by several approaches.[16–18,24] These studies exploited the mutant forms of CAR constructs, which bound to the same antigen with different affinities. In the current study, the MVR CAR-T system used a single CAR construct combined with LCL targets of various *HLA-DRB1* alleles. The former approaches have the potential to result in input variations among the CAR-Ts (e.g., transduction rate,[16] expression level,[24] and stability and tonic signal of CARs[25]), but not in the target cells. This feature is particularly useful in evaluating the affinity effect on target cells, such as *in vitro*/*in vivo* efficiency test, owing to the absence of inter-target variations (for example, growth rate, killing susceptibility, and *in vivo* graft rate of target cells). Accordingly, the advantage of the MVR CAR-T system lies in the evaluation of the cellular changes of CAR-T (e.g., polyfunctionality and gene expression), which is elicited differently by various affinities. Additionally, the use of a naturally evolved polymorphic antigen, HLA-DR, which covers a wide affinity range, can circumvent labor- and time-intensive processes for designing various CARs with broad affinity ranges.

The limitations of this system involve the presence of two *HLA-DRB1* alleles (and thus two HLA-DR antigens) in a target cell, which makes it difficult to define the exact K_D value as an affinity value to the target-cell antigen. In this current study, we used an SD_{50} value to compare the binding strengths against LCLs. Further efforts in designing an LCL possessing a single *HLA-DRB1* allele should be made to quantitatively interrogate the effects of the defined K_D on functional outcomes. Another concern is the interactions of HLA-DR with LAG3, TCR, and CD4 (particularly of $CD4^+$ CAR-T). This may potentially contribute to the activation capacity of $CD4^+$ MVR CAR-T. Of note, there were no significant differences in the affinity-associated polyfunctionality changes between $CD4^+$ and $CD8^+$ CAR-T (Figures 6A–6F), and LAG3 expression was not detected on MVR CAR-T (data not shown). However, the influence of these molecules should be carefully considered when evaluating the effects of an extremely weak MVR–HLA-DR affinity and the effect of a repeated stimulation, where LAG3 can be expressed during the assay. Finally, the effect of HLA-DR expression on various cells in PBMCs should be considered. During an MVR CAR-T generation, CAR-T interacts with HLA-DR on the dendritic cells, monocytes, B cells, and some activated T cells. This interaction may cause cellular changes in CAR-T, as has been reported in previous studies.[9,26] The high polyfunctionality observed in MVR CAR-T (compared to CD19 CAR-T; Figures 6A–6D) may be due to the increased effector

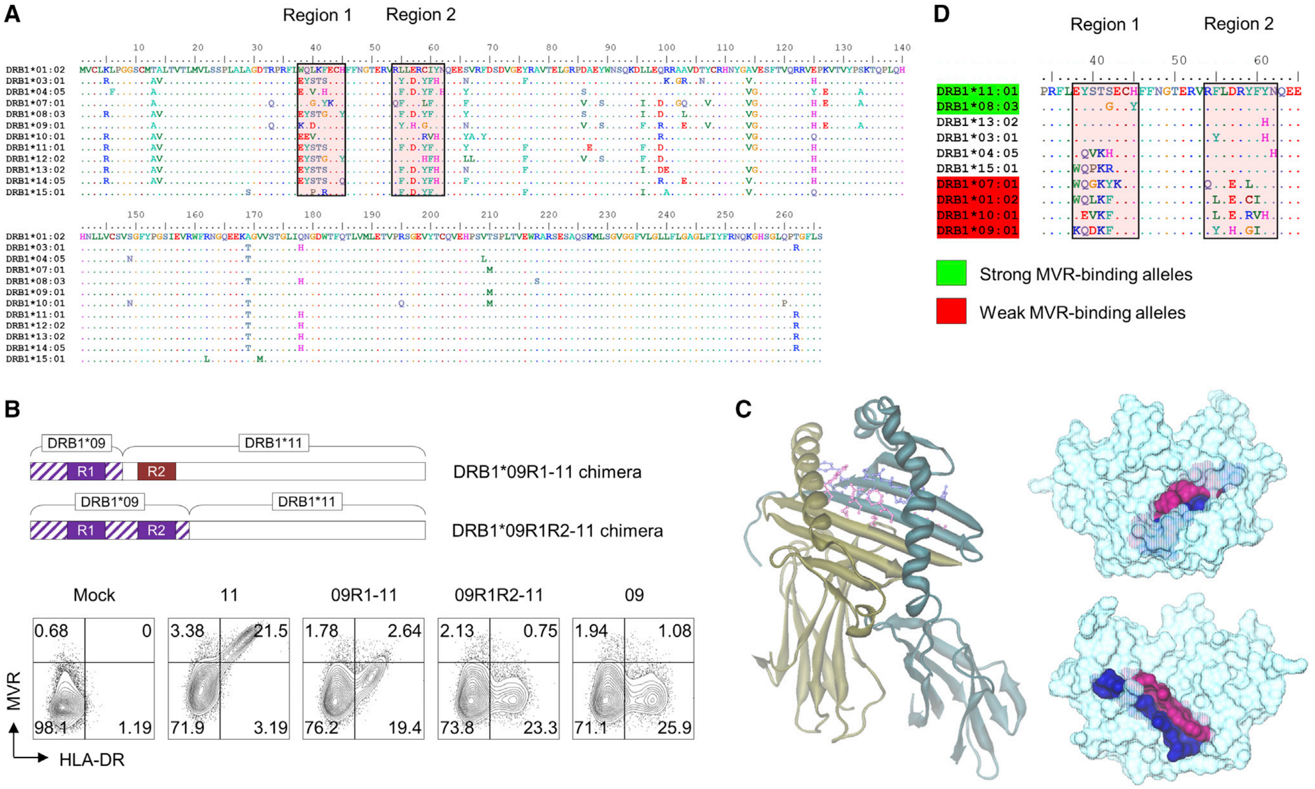

Figure 4. MVR Recognizes a Conformational Epitope in the Peptide-Binding Groove of HLA-DR

Identification of the MVR-binding epitope in the HLA-DR complex. (A) Twelve HLA-DRB1 variants were aligned based on their amino acid sequences. Two highly polymorphic regions are indicated. (B) The evaluation of MVR binding against the HLA-DR complex containing the HLA-DRB1 chimera. The upper drawing describes the constructs of two HLA-DRB1 chimeras that consist of different portions of HLA-DRB1*09:01 and HLA-DRB1*11:01. HLA-DRA-expressing dDR-CIITA cells were transfected with four HLA-DRB1-expressing vectors (HLA-DRB1*09:01, HLA-DRB1*11:01, and two chimeras) and analyzed for intact HLA-DR expression and MVR binding by flow cytometry. (C) The predicted MVR-binding epitope is highlighted in the reported HLA-DR structure (PDB: 3PGD).[14] A cartoon diagram (left) of the α chain (yellow) and β chain (cyan) of the HLA-DR complex is shown. Top (upper right) and bottom views (lower right) of the molecular surface representation are shown. For all images, region 1 (purple) and region 2 (blue) are indicated. (D) The alignment of the amino acid sequences around epitope regions. The HLA-DRB1 types evaluated in this study were aligned. Strong (green) and weak (red) MVR-binding alleles are highlighted.

functions that result from the MVR–HLA-DR interactions during the CAR-T generation process.

Several groups have investigated the relationship between CAR affinity and *in vitro* killing efficiency.[16–18] Chmielewski et al.[16] used the ErbB2-targeting CARs with affinities ranging from 320 nM to 0.015 nM (K_D), and demonstrated that the lowest affinity CAR-T (K_D of 320 nM) had a reduced *in vitro* killing efficiency and a higher affinity. The authors also observed similarly reduced anti-tumor efficacy of CAR-T *in vivo*. Drent et al.[17] designed the CD38-targeting CARs with a broad affinity range (K_D from >1 μM to 10 nM) and showed that CAR-Ts of micromolar affinities reduced the *in vitro*/*in vivo* killing efficiency, particularly against the CD38^{low} target cells. Park et al.[18] established the ICAM-1-targeting natural ligand-based CARs, with affinities ranging from 1.5 mM to 1 nM. Using the extremely broad affinity range, the authors demonstrated the clear correlation between the CAR affinity and a long-term *in vitro* killing efficiency. Interestingly, Arcangeli et al.[24] who used the CD123-targeting CARs of K_D spanning from 300 nM to 1 nM, did not observe a significant difference in the killing effect. This discrepancy may be due to the use of different target antigens and/or CAR constructs (e.g., 2nd generation versus 3rd generation CARs and 4-1BB versus CD28 costimulatory domain). In the current study, MVR affinities for three HLA-DRB1 types that showed strong, intermediate, and weak bindings were 88.1 nM (HLA-DRB1*11:01), 359 nM (HLA-DRB1*15:01), and below the detection limit (HLA-DRB1*09:01), respectively. Considering the complex nature of this system (i.e., the presence of two *HLA-DRB1* alleles in each LCL) and the results of biolayer interferometry analysis and the sigmoidal dose-binding curve (Table 2; Figure 5B), affinities of MVR to various LCLs may range from 10^{-6} M to 10^{-8} M. Therefore, this study revealed the affinity-associated functional characteristics of the 4-1BB-containing second-generation CAR-T within the K_D range. Noteworthy, CD19 CAR, which was compared with MVR CAR throughout this study, showed distinct functional patterns that could not be explained from an affinity that was observed to be extremely strong (K_D of

Table 2. Result Summary of Biolayer Interferometry Analysis

HLA-DRB1 Type	K_D (M)	K_D Error	k_a (1/Ms)	k_a Error	k_{dis} (1/s)	k_{dis} Error
11:01	8.81×10^{-8}	8.32×10^{-10}	1.33×10^{4}	9.66×10^{1}	1.17×10^{-3}	7.06×10^{-6}
15:01	3.59×10^{-7}	3.55×10^{-9}	7.63×10^{3}	7.01×10^{1}	2.74×10^{-3}	9.88×10^{-6}
09:01	not determined					

328 pM).[27] This discrepancy may be due to the differences in the antigen density, as lymphoma cell lines indicates a 10-fold higher surface expression of HLA-DR compared to that of CD19.[28] Additionally, the extent of a tonic signal, which is reduced in CD19 CAR compared to those in other CAR constructs,[25] may have an effect on the observed moderate effector-functions of CD19 CAR-T used in this study.

By using the 4-1BB-containing second-generation MVR CAR-T, we observed an affinity-dependent increase in the CAR-T killing efficiency (Figure 5E), implying the validity of the MVR CAR-T system in the CAR affinity studies. Interestingly, LCL8125, which did not show a significant MVR-scFv binding via flow cytometry analysis, was effectively killed by MVR CAR-T, which was similar to the result obtained in a previous study.[9] The inconsistency in assay results may be attributed to the fast dissociation rates of MVR-scFv (Table 2) and the CAR-intrinsic immune synapses that drive a rapid cytotoxicity.[29] This system enabled the evaluation of the effect of affinity on CAR-T polyfunctionality. We found that the increase in polyfunctionality was followed by a polyfunctional plateau phase during the escalation of CAR affinity (Figures 6E and 6F). Considering the importance of polyfunctional T cells in an anti-tumor immunity,[30–32] our results suggest that there is an affinity limit above which the therapeutic efficacy is not improved. To corroborate this, studies validating the effect of CAR affinity on *in vivo* anti-tumor efficacy need to be conducted in the future. Recent studies have suggested that AICD of CAR-T impairs the therapeutic efficacy by decreasing *in vivo* persistence.[19–21] The extent of AICD was affected by spacers,[19,20] costimulation,[21] and transmembrane domains.[33] Additionally, the changes in AICD were accompanied by an altered CAR-T signaling.[20,21] As CAR-T signaling has been strongly associated with the CAR affinity, we sought to investigate the effect of affinity on AICD of CAR-T using the MVR CAR-T system. We found that the proportion of the apoptotic CAR-T increased when CAR interacted with high-affinity ligands (Figures 6G–6I). Given that a low-affinity CAR enhances the CAR-T persistence in preclinical and clinical studies,[8,18,27] it is likely that the low CAR–target interactions and the resulting decrease in AICD (as shown in this study) is crucial for an effective CAR-T therapy. However, because the increase in CAR affinities also enhances the effector functions of CAR-T, the optimal affinity range where CAR-T induces sufficient immune responses while reducing AICD should be determined by further studies.

To conclude, the current system enables an effective evaluation of the affinity-associated functional profiles of CAR-T. We hope that this system will help to advance the understanding of the characteristics of T cell-engaging immunotherapies.

MATERIALS AND METHODS

Human Samples

This study was reviewed and approved by the Institutional Review Board of the National Cancer Center. Blood and bone marrow samples used in this study were collected from healthy donors and patients after obtaining appropriate informed consent at the National Cancer Center. Frozen sections of DLBCL were provided by the Catholic Research Tissue Specimen Bank (Seoul, Korea).

Commercial Antibodies

The commercial antibodies used in this study are as follows: anti-mouse immunoglobulin G (IgG): fluorescein isothiocyanate (FITC; #555988, polyclonal, BD Biosciences, San Jose, CA, USA), anti-mouse IgG:PE (#PA1-84395, polyclonal, Thermo Fisher Scientific, Waltham, MA, USA), anti-CD19:FITC (#345788, SJ25C1, BD Biosciences, San Jose, CA, USA), anti-mouse IgG:HRP (#P044701-2, polyclonal, Agilent Technologies, Santa Clara, CA, USA), anti-mouse IgG:Alexa Fluor 546 (#A-11030, polyclonal, Thermo Fisher Scientific, Waltham, MA, USA), anti-HLA-DR:FITC (#556643, G46-6, BD Biosciences, San Jose, CA, USA), anti-HLA-DR:PE (#555812, G46-6, BD Biosciences, San Jose, CA, USA), anti-HLA-DR-CLIP:FITC (#555981, Cer-CLIP, BD Biosciences, San Jose, CA, USA), anti-DYKDDDDK (FLAG):APC (#130-101-564, REA216, Miltenyi Biotec, Bergisch Gladbach, Germany), anti-CD19:PE (#340364, SJ25C1, BD Biosciences, San Jose, CA, USA), anti-CD8:FITC (#555366, RPA-T8, BD Biosciences, San Jose, CA, USA), anti-CD4:PE (#555347, RPA-T4, BD Biosciences, San Jose, CA, USA), anti-IFN-γ:PE-Cy7 (#557643, B27, BD Biosciences, San Jose, CA, USA), anti-TNF-α:PerCP-Cy5.5 (#560679, MAb11, BD Biosciences, San Jose, CA, USA), anti-IL-2:BV421 (#562914, 5344.111, BD Biosciences, San Jose, CA, USA), anti-MIP-1β:APC-H7 (#561280, D21-1351, BD Biosciences, San Jose, CA, USA), anti-CD107a:BV510 (#563078, H4A3, BD Biosciences, San Jose, CA, USA), anti-CD20:APC-H7 (#560734, 2H7, BD Biosciences, San Jose, CA, USA), purified human IgGs (#I2511, polyclonal, Sigma-Aldrich, St. Louis, MO, USA), purified mouse IgGs (#I8765, polyclonal, Sigma-Aldrich, St. Louis, MO, USA), and anti-DYKDDDDK(FLAG):biotin (#130-101-566, REA216, Miltenyi Biotec, Bergisch Gladbach, Germany).

Cells and Media

L3055 cells were maintained on a layer of HK cells in Iscove's modified Dulbecco's medium (#9032, FUJIFILM Irvine Scientific, Santa Ana, CA, USA) supplemented with 3 mM glutamine, 1% penicillin/streptomycin (#15140-122, Thermo Fisher Scientific, Waltham, MA, USA), and 10% heat inactivated fetal bovine serum (#FBS-BBT-5XM, Rocky Mountain Biologicals, Missoula, MT, USA).[34,35] RPMI1640 medium (R10+; #LM011-01, Welgene, Daejeon, Korea) or Dulbecco's modified Eagle's medium (D10+; #LM001-05, Welgene, Daejeon, Korea) was supplemented with 1% penicillin/streptomycin and 10% heat inactivated fetal bovine serum to maintain most of the cell lines used in this study. THP-1, 293,

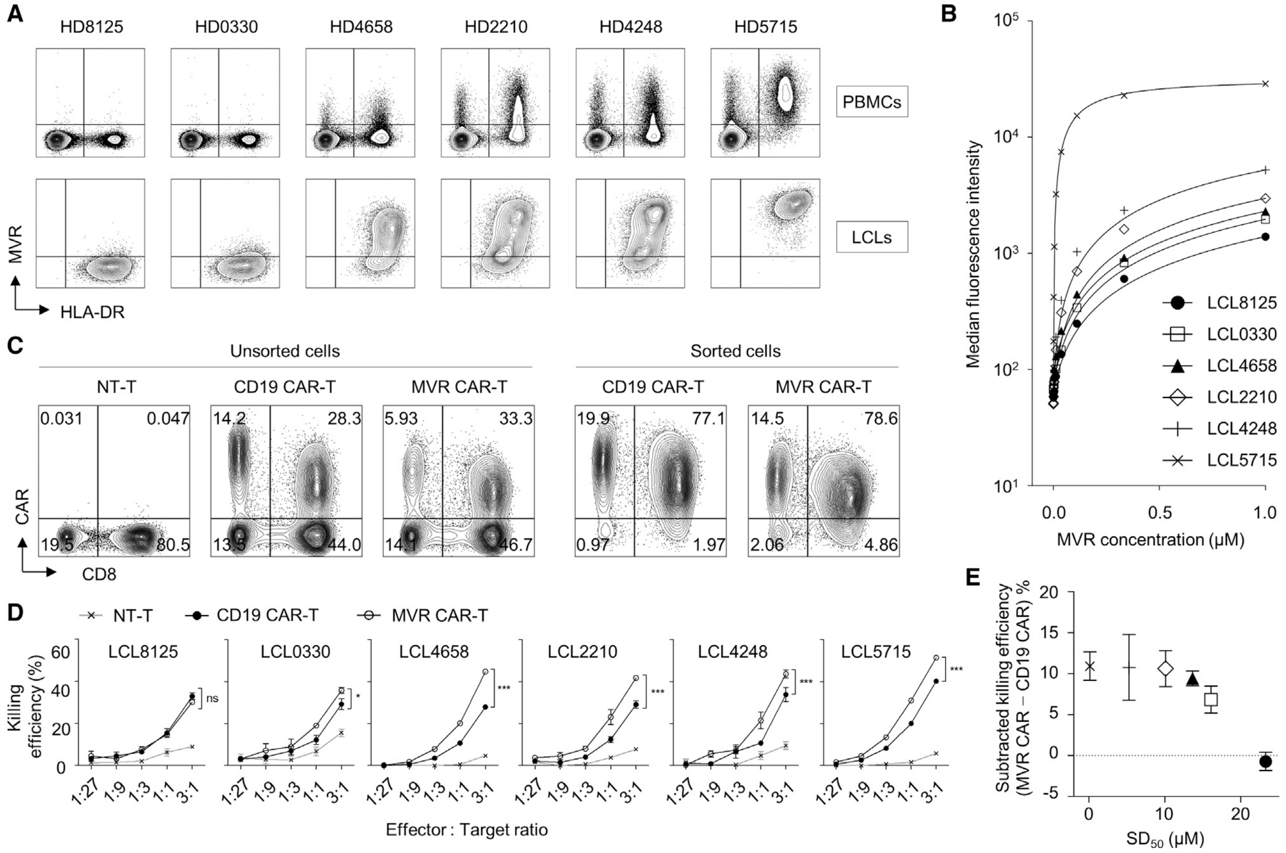

Figure 5. MVR/LCL Pairs of Various Binding Affinities Enabled the Functional Evaluation of CAR-Ts

The effect of affinity on CAR-T killing efficiency was evaluated using MVR-engineered CAR-Ts and LCLs of varying affinities. (A) The generation of target cells with various MVR-binding affinities. PBMCs derived from six donors were transformed with EBV to generate LCLs. All PBMCs and LCLs were co-stained with MVR and anti-HLA-DR and analyzed by flow cytometry. (B) Affinity evaluation of the generated LCLs. LCLs were stained with Alexa Fluor 647-conjugated MVR at the indicated concentrations and analyzed by flow cytometry. The median fluorescence intensity of each sample was determined and used to draw a sigmoidal binding curve. (C) The generation of effector T cells. T cells were transduced with second-generation CD19 CAR or MVR CAR-coding lentiviral vectors. Non-transduced T cells (NT-Ts) served as negative controls. CAR-expressing cells were magnetically sorted and used. (D) The evaluation of killing efficiency. Effector cells were co-incubated with target cells at the indicated effector to target ratio for 4 h, and the killing efficiency was calculated. n = 3 experimental replicates. ns, not significant; *$p < 0.05$; ***$p < 0.001$; two-way ANOVA. Error bars indicate means ± SD. (E) The relationship between killing efficiency and CAR affinity. For six target cells, the MVR concentration that yields half maximal binding (SD_{50}) in (B) was calculated. The differences between the killing efficiencies of MVR CAR-Ts and CD19 CAR-Ts (at an E:T ratio of 1:1) against each target cell were plotted with their corresponding SD_{50} values. Each symbol indicates the same LCLs in (B). n = 3 experimental replicates. Error bars indicate means ± SD.

PC3, Jurkat, A431, BC-1, 1A2, JVM-2, and B95-8 were purchased from the American Type Culture Collection and cultured in R10+ medium. SNU638, SNU20, and SNU538 were obtained from the KCLB (Korean Cell Line Bank, Seoul, Korea) and cultured in R10+ medium. The HeLa-CIITA cell line was kindly provided by Dr. Philippe Pierre (Centre d'Immunologie de Marseille-Luminy, Marseille, France) and cultured in D10+ medium. PBMCs were isolated from blood using conventional Ficoll gradient centrifugation, and then suspended in primary culture grade RPMI1640 medium (#LM011-77, Welgene, Daejeon, Korea) supplemented with 1% penicillin/streptomycin and 10% heat inactivated fetal bovine serum (pR10+) until use. LCLs were established by transforming PBMCs with EBV. In detail, the supernatant of exponentially growing B95-8 cells was harvested and filtered through a 0.45 μm filter. PBMCs were incubated in the EBV-containing supernatant with a volume of 5 mL/10^7 cells for 2 h at 37°C. The cells were transferred to a T75 flask and treated with 1 μg/mL cyclosporine A. After 3 weeks, the immortalized LCLs were checked for CD19 and HLA-DR expression and used in the experiments. All LCLs were cultured in R10+ medium. dDR-CIITA, the HLA-DRA-expressing cell line, was generated by interrupting the HLA-DRB1 locus in HeLa-CIITA cells. In detail, a pLCv2-DRB1 plasmid designed by Han et al.,[9] which co-expresses HLA-DRB1-targeting sgRNA and Cas9, was transfected into HeLa-CIITA using Lipofectamine 3000 (#L3000075, Thermo Fisher Scientific, Waltham, MA, USA). 1 week post-transfection, HeLa-CIITA cells were single-cell cloned by limiting dilution and screened for HLA-DR expression by flow cytometry. dDR-CIITA was cultured in D10+ medium. Lenti-X

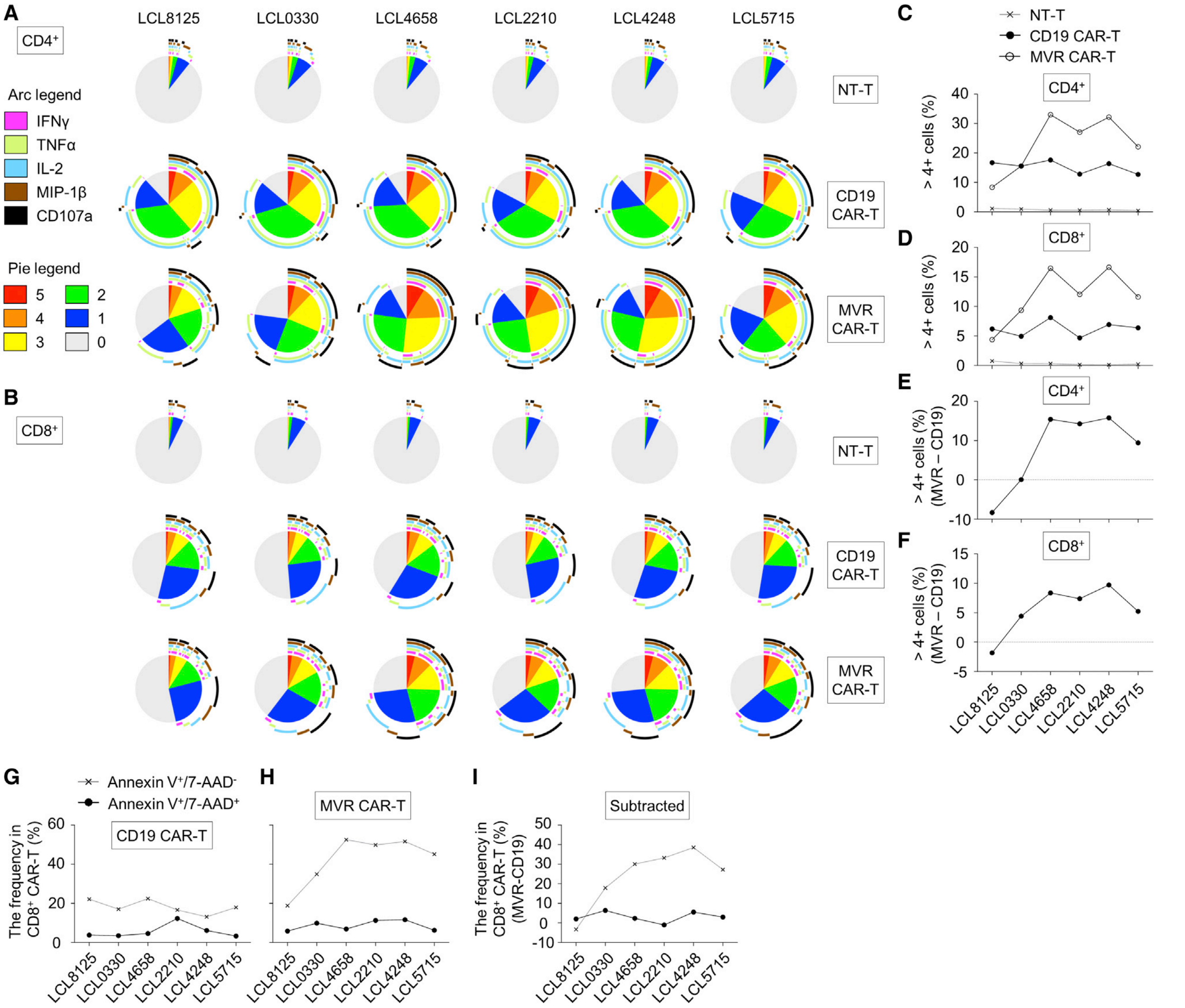

Figure 6. MVR CAR-T System Defines Polyfunctional Plateau of CAR-T in a High Affinity Range

The effect of affinities on CAR-T polyfunctionality was evaluated using MVR CAR-T system. (A and B) The polyfunctionality of CD19 CAR-Ts or MVR CAR-Ts stimulated with a panel of LCLs (which is shown in Figure 5A). Non-transduced T cells (NT-Ts) served as negative controls. The length of the arc of each color indicates the frequency of cells expressing the corresponding marker. The area of the pie of each color indicates the frequency of cells expressing the given number of functional markers. $CD4^+$ (A) and $CD8^+$ (B) cells were analyzed separately. (C and D) The frequency of NT-Ts, CD19 CAR-Ts, and MVR CAR-Ts expressing more than four functional markers. This frequency was determined based on the numeric data of polyfunctional analysis. $CD4^+$ (C) and $CD8^+$ (D) cells were analyzed separately. (E and F) The relationship between the affinity and polyfunctionality of MVR CAR-T. The frequency of CD19 CAR-Ts expressing more than four functional markers was subtracted from that of MVR CAR-Ts. The numerical data of (C) and (D) were used for the calculation. $CD4^+$ (E) and $CD8^+$ (F) cells were calculated separately. (G and H) The activation-induced cell death of CD19 CAR-Ts (G) or MVR CAR-Ts (H) stimulated with LCLs. The increase in the frequency of Annexin V^+/7-AAD^- apoptotic cells and Annexin V^+/7-AAD^+ dead cells was calculated. (I) The apoptotic/dead-cell frequency of CD19 CAR-Ts was subtracted from that of MVR CAR-Ts to eliminate inter-LCL variation. The numerical data of (G) and (H) were used for the calculation.

293T was purchased from Takara Bio (#632180, Shiga, Japan) and cultured in D10+ medium.

Antibody Preparation

6- to 8-week-old BALB/c female mice were purchased from Orient Bio (Seongnam, Korea) and used to generate antibodies. All mice were maintained under specific-pathogen-free conditions in the animal facilities of the National Cancer Center (Goyang, Korea). Procedures were approved by the Institutional Animal Care and Use Committee (IACUC) of the National Cancer Center Institute (NCCI). The NCCI animal facility is fully accredited by the Association for Assessment and Accreditation of Laboratory Animal Care

International (AAALAC International). Animal experiments were conducted following the Guidelines on the Care and Use of Laboratory Animals from the Institute of Laboratory Animal Resources (ILAR). Mice were intraperitoneally immunized twice 2 weeks apart with 2×10^7 L3055 cells, and intravenously injected with 1×10^7 L3055 cells 3 weeks later. Splenocytes were collected from the immunized mice 4 days after the final injection and fused with SP2/0 myeloma cells at a 3:1 ratio by slowly adding warmed polyethylene glycol 1500 solution (#10783641001, Sigma-Aldrich, St. Louis, MO, USA) to generate hybridomas. The hybridomas were plated onto 96-well plates at a density of 5×10^5 cells/well and cultured in HAT (hypoxanthine, aminopterin, and thymidine) selection medium for 14 days. To select B lymphoid cell-specific hybridoma clones, we applied the supernatant of each well to L3055 cells to analyze the binding capacity of secreted antibodies by flow cytometry. The wells containing L3055-specific antibodies were further single-cell cloned, and the specificity of each clone was evaluated by flow cytometry (Figure 1b). The isolated MVR clone was used as FLAG-tagged scFv,[9] full IgG, or Alexa Fluor 647-conjugated IgG in this study. The full IgG form of MVR was purified from the culture supernatant using Protein G Sepharose 4 Fast Flow (#17061802, GE Healthcare, Chicago, IL, USA) according to the manufacturer's protocol. FITC- or Alexa Fluor 647-conjugated MVR was generated using purified MVR IgG and a Pierce FITC Antibody Labeling Kit or an Alexa Fluor 647 Protein Labeling Kit (#53027 and #A20173, Thermo Fisher Scientific, Waltham, MA, USA).

Flow Cytometry

FACSCalibur and FACSVerse (BD Biosciences, San Jose, CA, USA) were used to acquire flow cytometry data in this study. All gating strategies used in this study are shown in Figure S2. For sample preparation, staining with commercial antibodies was performed according to the manufacturer's protocol. To stain cells with MVR-scFv (Figures 3A and 3B,4B, and 5A), we stained 1×10^6 cells with 1 μg Ni-nitrilotriacetic acid (NTA)-purified MVR-scFv for 30 min at 4°C, then washed them once and further stained them with anti-FLAG:APC according to the manufacturer's protocol.[9] MVR-containing culture medium was used to screen the binding capacities of isolated antibodies (Figure 1B). Hybridomas (including MVR) were cultured in D10+ medium for 7 days to maximize antibody production. Half a million target cells were stained with 200 μL of hybridoma culture medium for 30 min at 4°C, washed once, and further stained with 0.5 μg of anti-mouse IgG:FITC. The stained cell lines were washed twice and analyzed. ALL/CLL patient-derived primary cells were evaluated for MVR-binding using FITC-conjugated MVR IgG (Figure 1C). One million cells were co-stained with 1 μg anti-CD19:PE and FITC-conjugated MVR, washed twice, and analyzed. Alexa Fluor 647-conjugated MVR was used to compare MVR-binding affinities among LCLs (Figure 5B). In detail, 1×10^5 LCLs (>80% viability) were counted using ADAM-MC2 (NanoEnTek, Seoul, Korea) and incubated with 3-fold step-diluted concentrations of antibody ranging from 1 μM to 152 pM for 30 min at 4°C. The LCLs were then washed twice and analyzed for MVR binding by flow cytometry. A sigmoidal dose-binding curve was drawn based on the measured median fluorescence intensity and the corresponding antibody concentration. An SD_{50} value was calculated for each LCL from the sigmoidal dose-binding curve using Prism (GraphPad Software, San Diego, CA, USA). The robust fitting method in the one site (total binding) model was used to calculate the SD_{50} values, and the maximum binding was assumed to be shared among all LCLs. The polyfunctionality of CAR-T was determined according to a previous study.[9] Briefly, LCLs were labeled using a CellTrace carboxyfluorescein succinimidyl ester cell proliferation kit (#C34554, Thermo Fisher Scientific, Waltham, MA, USA) and co-cultured with T cells at a T cell:LCL ratio of 3:1 for 6 h in the presence of a protein transport inhibitor cocktail (#00-4980, Thermo Fisher Scientific, Waltham, MA, USA) and anti-CD107a:BV510. The cells were stained with anti-CD4:PE and the fixable viability dye eFluor 660 (#65-0864, Thermo Fisher Scientific, Waltham, MA, USA), and stained intracellularly with anti-IFN-γ:PE-Cy7, anti-TNF-α:PerCP-Cy5.5, anti-IL-2:BV421, and anti-MIP-1β:APC-H7 using a cytofix/cytoperm fixation/permeabilization kit (#554714, BD Biosciences, San Jose, CA, USA) based on the manufacturer's protocols. The stained samples were analyzed by flow cytometry and the frequency of cells expressing multiple markers was determined according to the Boolean combination gating analysis (Figure S2E). The data were used to draw pie charts using SPICE v6.0 software. The AICD of CAR-T was measured by staining with Annexin V:BV421 (#563973, BD Biosciences, San Jose, CA, USA) and 7-AAD (#559925, BD Biosciences, San Jose, CA, USA). Specifically, a total of 4×10^5 T cells/LCLs were co-cultured at a T cell:LCL ratio of 3:1 for 6 h in the presence of anti-CD8:FITC and anti-CD20:APC-H7 in a flat-bottom 96-well plate. The cells were collected and stained with Annexin V:BV421 and 7-AAD according to the manufacturer's protocols. The stained samples were analyzed by flow cytometry, and the frequency of cells positive for Annexin V:BV421 and/or 7-AAD fluorescence in CD20-negative/CD8-positive CAR-T was determined. To calculate the relative change in the frequency of apoptotic/dead cells during LCL stimulation, we subtracted the proportion of Annexin V^+/7-AAD^+ or Annexin V^+/7-AAD^- in unstimulated CAR-T from that of the LCL-stimulated CAR-T.

Microscopy

For immunohistochemistry of tissues from DLBCL patients, frozen sections were pre-incubated with purified human IgGs. The tissues were then incubated with 10 μg/mL purified MVR IgG for 2 h at 8°C–12°C and stained with anti-mouse IgG-HRP. For color development, the tissues were stained with 3,3′-diaminobenzidine and counterstained with hematoxylin. All samples were mounted with VectaMount Permanent Mounting Medium (#H-5000, Vector Laboratories, Burlingame, CA, USA), and images were taken under a light microscope. For immunofluorescence analysis of LCL5715, cells were stained with MVR and further stained with anti-mouse IgG:Alexa Fluor 546. To saturate anti-mouse IgG binding, we washed the cells with PBS (10 mM phosphate, 137 mM NaCl, 3.7 mM KCl, pH 7.2) containing 0.1% BSA and incubated them with 1 μg/mL polyclonal mouse IgGs for 30 min at 4°C. The cells were further stained with anti-HLA-DR:FITC and fixed with 4% paraformaldehyde. The

surface-stained cells were attached to poly-L-lysine-coated glass slides using a CellSpin spinner system (Hanil Scientific, Gimpo, Korea) and visualized with a confocal microscope, the LSM 510 Meta (Zeiss, Oberkochen, Germany).

Mass Spectrometry

For the isolation of MVR-binding protein, 1 mL Protein A/G PLUS-Agarose (#sc-2003, Santa Cruz Biotechnology, Dallas, TX, USA) was saturated with 10 mg purified MVR IgG and cross-linked with disuccinimidyl suberate. The cross-linked resin was packed into columns and washed with PBS. LCL5715 cells (2×10^8 cells) suspended in Tris-buffered saline (50 mM, pH 7.5 Tris, and 150 mM NaCl; TBS) containing 1% Triton X-100 were transferred to a glass homogenizer and disrupted with 12 pestle strokes. Cell debris was removed by centrifugation and the lysate was loaded onto affinity columns. The column was washed with TBS and the bound protein was eluted with 100 mM glycine solution (pH 3.0). The eluant was concentrated using trichloroacetic acid precipitation and separated on a 12% SDS-PAGE gel. The gel was silver-stained with a SilverSNAP Stain Kit (Thermo Fisher Scientific, Waltham, MA, USA) according to the manufacturer's protocol. The untrimmed image is shown in Figure S3. Tandem mass spectrometry (MS/MS) of each protein band was performed using nano-ESI on a Q-TOF2 mass spectrometer (AB SCIEX) at Proteinworks (Daejeon, Korea). In detail, proteins were subjected to in-gel trypsin digestion.[36] To desalt and concentrate the peptide mixture, we used a custom-made chromatographic column packed with 100–300 nL of POROS reverse phase R2 (20–30 μm bead size, PerSeptive Biosystems, Framingham, MA, USA) prior to mass spectrometric analysis. The column was loaded with the digested peptide mixture and washed with 30 μL of 5% formic acid. Peptides were then eluted with 1.5 μL of 50% methanol/49% H_2O/1% formic acid directly into a nano-electrospray needle and subjected to mass analysis. The ionization voltage was 1 kV, and the nitrogen backpressure was set at 0–5 psi to produce a stable flow rate (10–30 nL/min). The source temperature was room temperature, and the cone voltage was 40 V. A quadrupole analyzer was used to select precursor ions for fragmentation in the hexapole collision cell. The collision gas was Ar ($6–7 \times 10{-}5$ mbar) and the collision energy was 25–40 V. Product ions were analyzed using an orthogonal TOF analyzer fitted with a reflector, a micro-channel plate detector, and a time-to-digital converter. The data were processed using a peptide sequence system (AB SCIEX).

ELISA

Streptavidin type II (1 μg/mL in 100 μL PBS, Fujifilm-Wako, Osaka, Japan) was immobilized in transparent microplate wells (Greiner 655061, Tokyo, Japan) at 4°C overnight. The wells were blocked with 20% Immunoblock (DS Pharma, Osaka, Japan) and washed three times with PBST (PBS containing 0.1% Tween 20). HLA-DRB1*11:01, HLA-DRB1*15:01, or HLA-DRB1*09:01 complexed with biotinylated HLA-DRA*01:01 and CLIP (4 μg/mL; ProImmune, Oxford, UK) in 100 μL PBS was added and incubated for 1 h at 25°C. The wells were then washed three times with PBST and incubated with purified C-terminally StreptagII-fused MVR-scFv (1 μg/mL in 100 μL PBS) or PBS for 1 h at 25°C. After washing with PBST, 5,000-fold diluted Streptactin:HRP (BioRad, Hercules, CA, USA) was applied and incubated for 30 min at 25°C. The wells were washed with PBST and measured for HRP activity using a substrate (100 μg/mL 3,3′5,5′-tetramethylbenzidine and 0.02% H_2O_2 in 100 mM sodium acetate, pH 6.0).

Epitope Identification

The MVR-binding epitope was identified through four steps: (1) MVR-binding check and *HLA-DRB1* allele typing, (2) estimation of the potential epitope region, (3) epitope confirmation by chimera protein, and (4) verification of the epitope region in the protein structure. To examine the effect of the *HLA-DRB1* allele type on MVR binding, we subjected several cell lines and healthy volunteer-derived PBMCs to low resolution (2-digit; sequence-specific primer amplification method) or high resolution (6-digit; PCR-sequencing based typing) *HLA-DRB1* typing at Samkwang Medical Laboratories (Seoul, Korea). To estimate the potential epitope region, we aligned amino acid sequences of major HLA-DRB1 types from the IPD-IMGT/HLA database using BioEdit.[37] Two hypervariable regions identified by the alignment were further evaluated for their effect on MVR-binding using HLA-DRB1 chimeras. In detail, *HLA-DRB1*11:01:01*- and *HLA-DRB1*09:01:02*-coding mRNAs were isolated from LCL5778 and LCL8125 using an RNeasy Plus Mini Kit (#74136, QIAGEN, Hilden, Germany). The mRNAs were reverse transcribed using a SuperScript IV First-Strand Synthesis System (#18091050, Thermo Fisher Scientific, Waltham, MA, USA), and the coding genes were inserted into the pcDNA3.1 expression vector (#V79020, Thermo Fisher Scientific, Waltham, MA, USA) using conventional molecular biology techniques (pcDNA3.1_DRB1*110101 and pcDNA3.1_DRB1*090102). To generate partial *HLA-DRB1*09:01:02* chimera-expressing vectors that span the first hypervariable region (pcDNA3.1_DRB1*09R1-11) or the second hypervariable region (pcDNA3.1_DRB1*09R1R2-11), we used conventional overlap extension PCR technique. dDR-CIITA, the HLA-DRA-expressing cell line, was transfected with the generated HLA-DRB1 vectors using Lipofectamine 3000 according to the manufacturer's protocol. 2 days post transfection, dDR-CIITA was detached from the plate using PBS containing 5 mM EDTA and examined for HLA-DR expression and MVR-binding capacity. The localization of the two hypervariable regions (i.e., epitope regions) was displayed in the 3D protein structure. DRα and DRβ from the previously determined HLA-DR–CLIP structure (PDB: 3PGD) were used.[14] All structure images were rendered using VMD 1.9.3.

Biolayer Interferometry Analysis

The affinities between MVR-scFv and HLA-DR constructs were evaluated by using Octet K2 (Pall FortéBio, Fremont, CA, USA). HLA-DRB1*11:01, HLA-DRB1*15:01, or HLA-DRB1*09:01 types were complexed with biotinylated HLA-DRA*01:01 and CLIP was diluted in the K buffer (PBS supplemented with 0.1% BSA, 0.002% Tween20) and used for an HLA-DR immobilization. A streptavidin biosensor was incubated with 4 μg/mL of the HLA-DR protein and quenched with 10 μg/mL biotin in the K buffer. Typical immobilization levels were 1.8 nm, 1.0 nm, and 0.67 nm for HLA-DRB1*11:01, HLA-DRB1*15:01, and HLA-DRB1*09:01,

respectively. The corresponding affinities were determined by performing association and dissociation measurements at five concentrations between 2000 nM and 62.5 nM with double references (without HLA-DR and/or MVR-scFv). Samples were diluted in the K buffer. The association and dissociation phases were measured for 300 s. Data was fitted to a 1:1 interaction model using a Data Analysis 8.1 HD software (Pall FortéBio).

CAR-T Generation and Killing Assay

The CD19-specific antibody and MVR-engineered CAR-Ts were generated according to the previously reported protocol.[9] In brief, Lenti-X 293T cells were co-transfected with three accessory plasmids (pMDLg/pRRE, pRSV-rev, and pMD.G) and a transfer vector, either pELPS-FLAG19BBz or pELPS-FLAGMVRBBz. 2 days post-transfection, the supernatant was harvested, filtered using a 0.45-μm filter, and concentrated using ultracentrifugation. To transduce T cells, we activated HD8125-derived PBMCs using a T Cell Activation/Expansion Kit (#130-091-441, Miltenyi Biotec, Bergisch Gladbach, Germany) and transduced with CAR-coding lentivirus (3 transduction units/cell). CAR-Ts were then expanded in R10+ medium containing recombinant human IL-2 (200 IU/mL). Following 14 days of expansion, CAR-positive cells were magnetically sorted using anti-FLAG:biotin and anti-Biotin MicroBeads UltraPure (#130-105-637, Miltenyi Biotec, Bergisch Gladbach, Germany) and used in this study. The killing efficiency of CAR-Ts was evaluated using a CytoTox-Glo Cytotoxicity Assay Kit (#G9291, Promega, Madison, WI, USA). In detail, 5×10^4 target cells (LCLs) in R10+ medium were applied to a Corning 96 well black polystyrene microplate (#CLS3603, Sigma-Aldrich, St. Louis, MO, USA). Effector cells (NT-T, CD19 CAR-T, and MVR CAR-T) were then added to the wells at effector to target ratios of 1:27, 1:9, 1:3, 1:1, or 3:1 and co-cultured for 4 h at 37°C. Following incubation, 50 μL of CytoTox-Glo Cytotoxicity Assay Reagent was added to each well, and the resulting luminescence signal was measured using TECAN infinite PRO 200 (Tecan Group, Männedorf, Switzerland). Wells containing only target cells and Digitonin-treated target cells served as background and maximum killing controls, respectively. The killing efficiency was calculated using the following equation: (sample signal − background signal)/maximum killing signal. To evaluate affinity-related killing activity, we determined the difference between the killing efficiencies of CD19 CAR-Ts and MVR CAR-Ts at an effector to target ratio of 1:1 among LCLs owing to the saturated killing activity at the highest effector to target ratio.

Statistics

All statistical tests in this study were performed using Prism (GraphPad Software, San Diego, CA, USA). A two-tailed unpaired Student's t test was used to determine significant differences between groups for the ELISA (Figure 3C). A two-way ANOVA was used to determine significant differences among groups for the killing efficiency test (Figure 5D). For all statistical tests, significance is indicated with asterisks (ns, not significant; *$p < 0.05$; **$p < 0.01$; ***$p < 0.001$).

SUPPLEMENTAL INFORMATION

Supplemental Information can be found online at https://doi.org/10.1016/j.omto.2020.04.004.

AUTHOR CONTRIBUTIONS

C.H. and B.K.C. conceived the project and contributed to experimental design and subsequent analysis. C.H. conducted the experiments related to the identification of the MVR antigen and epitope. B.K.C. conducted the experiments related to the development of antibody clones. S.-J.S. generated CAR-T. S.-H.K. contributed to flow cytometry, the evaluation of polyfunctionality, and AICD. S.H. and B.P. assisted with the identification of the MVR epitope. Y.H.K. generated EBV. H.-S.E. contributed to the identification of the MVR antigen and the selection of patient samples. Y.T., M.T., T.K., and H.U. contributed to the experimental design and data interpretation of the ELISA and biolayer interferometry analysis. B.S.K. supervised the project. C.H., B.K.C., S.-H.K., and B.S.K. wrote the manuscript.

CONFLICTS OF INTEREST

B.S.K. is the founder and Chief Executive Officer of Eutilex. The remaining authors declare no competing interests.

ACKNOWLEDGMENTS

We thank Dr. Philippe Pierre for providing the HeLa-CIITA cell line. We also thank Dr. Yong Sung Choi for providing the L3055 and HK cell lines. This work was supported by the National Research Foundation of Korea (NRF) grant funded by the Korean government (2018R1A6A3A01011692 to S.-H.K. from MOE and 2019R1C1C1008999 to C.H. from MSIT); the National Cancer Center of Korea (NCC-1810102/1910050/1911261 to B.K.C. and NCC-2010190 to C.H.); Japan Society for the Promotion of Science (JSPS KAKENHI grant, JP18H03851 to H.U.); Japan Science and Technology Agency (JST-Mirai grant, JPMJMI18D9 to H.U.); and the Korean Ministry of Trade, Industry, and Energy (GLOBAL R&D PROJECT, N0000901 to B.S.K.).

REFERENCES

1. Weiner, L.M., Surana, R., and Wang, S. (2010). Monoclonal antibodies: versatile platforms for cancer immunotherapy. Nat. Rev. Immunol. *10*, 317–327.

2. Weiner, L.M., Murray, J.C., and Shuptrine, C.W. (2012). Antibody-based immunotherapy of cancer. Cell *148*, 1081–1084.

3. Zhukovsky, E.A., Morse, R.J., and Maus, M.V. (2016). Bispecific antibodies and CARs: generalized immunotherapeutics harnessing T cell redirection. Curr. Opin. Immunol. *40*, 24–35.

4. Barrett, D.M., Teachey, D.T., and Grupp, S.A. (2014). Toxicity management for patients receiving novel T-cell engaging therapies. Curr. Opin. Pediatr. *26*, 43–49.

5. Kroschinsky, F., Stölzel, F., von Bonin, S., Beutel, G., Kochanek, M., Kiehl, M., and Schellongowski, P.; Intensive Care in Hematological and Oncological Patients (iCHOP) Collaborative Group (2017). New drugs, new toxicities: severe side effects of modern targeted and immunotherapy of cancer and their management. Crit. Care *21*, 89.

6. Neelapu, S.S., Tummala, S., Kebriaei, P., Wierda, W., Gutierrez, C., Locke, F.L., Komanduri, K.V., Lin, Y., Jain, N., Daver, N., et al. (2018). Chimeric antigen receptor T-cell therapy - assessment and management of toxicities. Nat. Rev. Clin. Oncol. *15*, 47–62.

7. Caruso, H.G., Hurton, L.V., Najjar, A., Rushworth, D., Ang, S., Olivares, S., Mi, T., Switzer, K., Singh, H., Huls, H., et al. (2015). Tuning sensitivity of CAR to EGFR density limits recognition of normal tissue while maintaining potent antitumor activity. Cancer Res. *75*, 3505–3518.

8. Liu, X., Jiang, S., Fang, C., Yang, S., Olalere, D., Pequignot, E.C., Cogdill, A.P., Li, N., Ramones, M., Granda, B., et al. (2015). Affinity-tuned ErbB2 or EGFR chimeric antigen receptor T cells exhibit an increased therapeutic index against tumors in mice. Cancer Res. *75*, 3596–3607.

9. Han, C., Sim, S.J., Kim, S.H., Singh, R., Hwang, S., Kim, Y.I., Park, S.H., Kim, K.H., Lee, D.G., Oh, H.S., et al. (2018). Desensitized chimeric antigen receptor T cells selectively recognize target cells with enhanced antigen expression. Nat. Commun. *9*, 468.

10. Slaga, D., Ellerman, D., Lombana, T.N., Vij, R., Li, J., Hristopoulos, M., Clark, R., Johnston, J., Shelton, A., Mai, E., et al. (2018). Avidity-based binding to HER2 results in selective killing of HER2-overexpressing cells by anti-HER2/CD3. Sci. Transl. Med *10*, eaat5775.

11. Chapman, C.J., Zhou, J.X., Gregory, C., Rickinson, A.B., and Stevenson, F.K. (1996). VH and VL gene analysis in sporadic Burkitt's lymphoma shows somatic hypermutation, intraclonal heterogeneity, and a role for antigen selection. Blood *88*, 3562–3568.

12. Fortin, J.S., Cloutier, M., and Thibodeau, J. (2013). Exposing the specific roles of the invariant chain isoforms in shaping the MHC class II peptidome. Front. Immunol. *4*, 443.

13. Robinson, J., Halliwell, J.A., Hayhurst, J.D., Flicek, P., Parham, P., and Marsh, S.G. (2015). The IPD and IMGT/HLA database: allele variant databases. Nucleic Acids Res. *43*, D423–D431.

14. Günther, S., Schlundt, A., Sticht, J., Roske, Y., Heinemann, U., Wiesmüller, K.H., Jung, G., Falk, K., Rötzschke, O., and Freund, C. (2010). Bidirectional binding of invariant chain peptides to an MHC class II molecule. Proc. Natl. Acad. Sci. USA *107*, 22219–22224.

15. Hunter, S.A., and Cochran, J.R. (2016). Cell-binding assays for determining the affinity of protein-protein interactions: technologies and considerations. Methods Enzymol. *580*, 21–44.

16. Chmielewski, M., Hombach, A.A., and Abken, H. (2011). CD28 cosignalling does not affect the activation threshold in a chimeric antigen receptor-redirected T-cell attack. Gene Ther. *18*, 62–72.

17. Drent, E., Themeli, M., Poels, R., de Jong-Korlaar, R., Yuan, H., de Bruijn, J., Martens, A.C.M., Zweegman, S., van de Donk, N.W.C.J., Groen, R.W.J., et al. (2017). A rational strategy for reducing on-target off-tumor effects of CD38-chimeric antigen receptors by affinity optimization. Mol. Ther. *25*, 1946–1958.

18. Park, S., Shevlin, E., Vedvyas, Y., Zaman, M., Park, S., Hsu, Y.S., Min, I.M., and Jin, M.M. (2017). Micromolar affinity CAR T cells to ICAM-1 achieves rapid tumor elimination while avoiding systemic toxicity. Sci. Rep. *7*, 14366.

19. Hudecek, M., Sommermeyer, D., Kosasih, P.L., Silva-Benedict, A., Liu, L., Rader, C., Jensen, M.C., and Riddell, S.R. (2015). The nonsignaling extracellular spacer domain of chimeric antigen receptors is decisive for in vivo antitumor activity. Cancer Immunol. Res. *3*, 125–135.

20. Künkele, A., Johnson, A.J., Rolczynski, L.S., Chang, C.A., Hoglund, V., Kelly-Spratt, K.S., and Jensen, M.C. (2015). Functional tuning of CARs reveals signaling threshold above which CD8þ CTL antitumor potency is attenuated due to cell Fas–FasL-dependent AICD. Cancer Immunol. Res. *3*, 368–379.

21. Gomes-Silva, D., Mukherjee, M., Srinivasan, M., Krenciute, G., Dakhova, O., Zheng, Y., Cabral, J.M.S., Rooney, C.M., Orange, J.S., Brenner, M.K., and Mamonkin, M. (2017). Tonic 4-1BB costimulation in chimeric antigen receptors impedes T cell survival and is vector-dependent. Cell Rep. *21*, 17–26.

22. Lin, T.S., Stock, W., Xu, H., Phelps, M.A., Lucas, M.S., Guster, S.K., Briggs, B.R., Cheney, C., Porcu, P., Flinn, I.W., et al. (2009). A phase I/II dose escalation study of apolizumab (Hu1D10) using a stepped-up dosing schedule in patients with chronic lymphocytic leukemia and acute leukemia. Leuk. Lymphoma *50*, 1958–1963.

23. Schweighofer, C.D., Tuchscherer, A., Sperka, S., Meyer, T., Rattel, B., Stein, S., Ismail, S., Elter, T., Staib, P., Reiser, M., and Hallek, M. (2012). Clinical safety and pharmacological profile of the HLA-DR antibody 1D09C3 in patients with B cell chronic lymphocytic leukemia and lymphoma: results from a phase I study. Cancer Immunol. Immunother. *61*, 2367–2373.

24. Arcangeli, S., Rotiroti, M.C., Bardelli, M., Simonelli, L., Magnani, C.F., Biondi, A., Biagi, E., Tettamanti, S., and Varani, L. (2017). Balance of anti-CD123 chimeric antigen receptor binding affinity and density for the targeting of acute myeloid leukemia. Mol. Ther. *25*, 1933–1945.

25. Long, A.H., Haso, W.M., Shern, J.F., Wanhainen, K.M., Murgai, M., Ingaramo, M., Smith, J.P., Walker, A.J., Kohler, M.E., Venkateshwara, V.R., et al. (2015). 4-1BB costimulation ameliorates T cell exhaustion induced by tonic signaling of chimeric antigen receptors. Nat. Med. *21*, 581–590.

26. Mamonkin, M., Rouce, R.H., Tashiro, H., and Brenner, M.K. (2015). A T-cell-directed chimeric antigen receptor for the selective treatment of T-cell malignancies. Blood *126*, 983–992.

27. Ghorashian, S., Kramer, A.M., Onuoha, S., Wright, G., Bartram, J., Richardson, R., Albon, S.J., Casanovas-Company, J., Castro, F., Popova, B., et al. (2019). Enhanced CAR T cell expansion and prolonged persistence in pediatric patients with ALL treated with a low-affinity CD19 CAR. Nat. Med. *25*, 1408–1414.

28. Schubert, I., Kellner, C., Stein, C., Kügler, M., Schwenkert, M., Saul, D., Stockmeyer, B., Berens, C., Oduncu, F.S., Mackensen, A., and Fey, G.H. (2012). A recombinant triplebody with specificity for CD19 and HLA-DR mediates preferential binding to antigen double-positive cells by dual-targeting. MAbs *4*, 45–56.

29. Davenport, A.J., Cross, R.S., Watson, K.A., Liao, Y., Shi, W., Prince, H.M., Beavis, P.A., Trapani, J.A., Kershaw, M.H., Ritchie, D.S., et al. (2018). Chimeric antigen receptor T cells form nonclassical and potent immune synapses driving rapid cytotoxicity. Proc. Natl. Acad. Sci. USA *115*, E2068–E2076.

30. Yuan, J., Gnjatic, S., Li, H., Powel, S., Gallardo, H.F., Ritter, E., Ku, G.Y., Jungbluth, A.A., Segal, N.H., Rasalan, T.S., et al. (2008). CTLA-4 blockade enhances polyfunctional NY-ESO-1 specific T cell responses in metastatic melanoma patients with clinical benefit. Proc. Natl. Acad. Sci. USA *105*, 20410–20415.

31. Ding, Z.C., Huang, L., Blazar, B.R., Yagita, H., Mellor, A.L., Munn, D.H., and Zhou, G. (2012). Polyfunctional $CD4^+$ T cells are essential for eradicating advanced B-cell lymphoma after chemotherapy. Blood *120*, 2229–2239.

32. Franzese, O., Palermo, B., Di Donna, C., Sperduti, I., Ferraresi, V., Stabile, H., Gismondi, A., Santoni, A., and Nisticò, P. (2016). Polyfunctional Melan-A-specific tumor-reactive CD8(+) T cells elicited by dacarbazine treatment before peptide-vaccination depends on AKT activation sustained by ICOS. OncoImmunology *5*, e1114203.

33. Alabanza, L., Pegues, M., Geldres, C., Shi, V., Wiltzius, J.J.W., Sievers, S.A., Yang, S., and Kochenderfer, J.N. (2017). Function of novel anti-CD19 chimeric antigen receptors with human variable regions is affected by hinge and transmembrane domains. Mol. Ther. *25*, 2452–2465.

34. Choe, J., Li, L., Zhang, X., Gregory, C.D., and Choi, Y.S. (2000). Distinct role of follicular dendritic cells and T cells in the proliferation, differentiation, and apoptosis of a centroblast cell line, L3055. J. Immunol. *164*, 56–63.

35. Kim, H.S., Zhang, X., and Choi, Y.S. (1994). Activation and proliferation of follicular dendritic cell-like cells by activated T lymphocytes. J. Immunol. *153*, 2951–2961.

36. Shevchenko, A., Wilm, M., Vorm, O., and Mann, M. (1996). Mass spectrometric sequencing of proteins silver-stained polyacrylamide gels. Anal. Chem. *68*, 850–858.

37. Robinson, J., Halliwell, J.A., McWilliam, H., Lopez, R., and Marsh, S.G. (2013). IPD–the Immuno Polymorphism Database. Nucleic Acids Res. *41*, D1234–D1240.

OMTO, Volume *17*

Supplemental Information

Polymorphic Region-Specific Antibody for Evaluation of Affinity-Associated Profile of Chimeric Antigen Receptor

Chungyong Han, Beom K. Choi, Seon-Hee Kim, Su-Jung Sim, Seongeun Han, Bomi Park, Yohei Tsuchiya, Masaki Takahashi, Young H. Kim, Hyeon-Seok Eom, Tetsuya Kitaguchi, Hiroshi Ueda, and Byoung S. Kwon

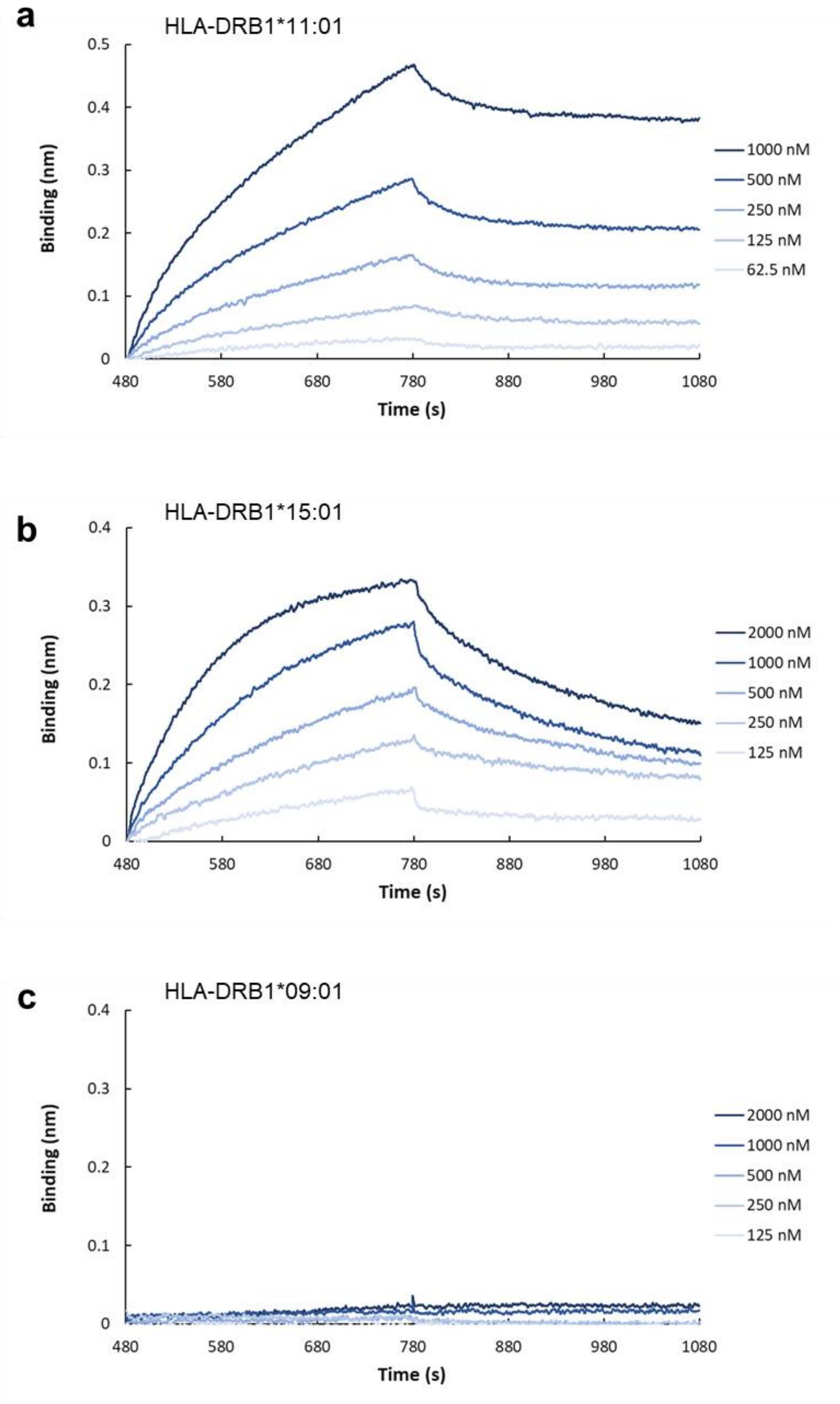

Supplementary Figure 1. The result of biolayer interferometry analysis. Protein interaction between immobilized HLA-DR (HLA-DRB1*11:01 [**a**], HLA-DRB1*15:01 [**b**] or HLA-DRB1*09:01 [**c**] complexed with HLA-DRA*01:01 and CLIP) and MVR-scFv was measured.

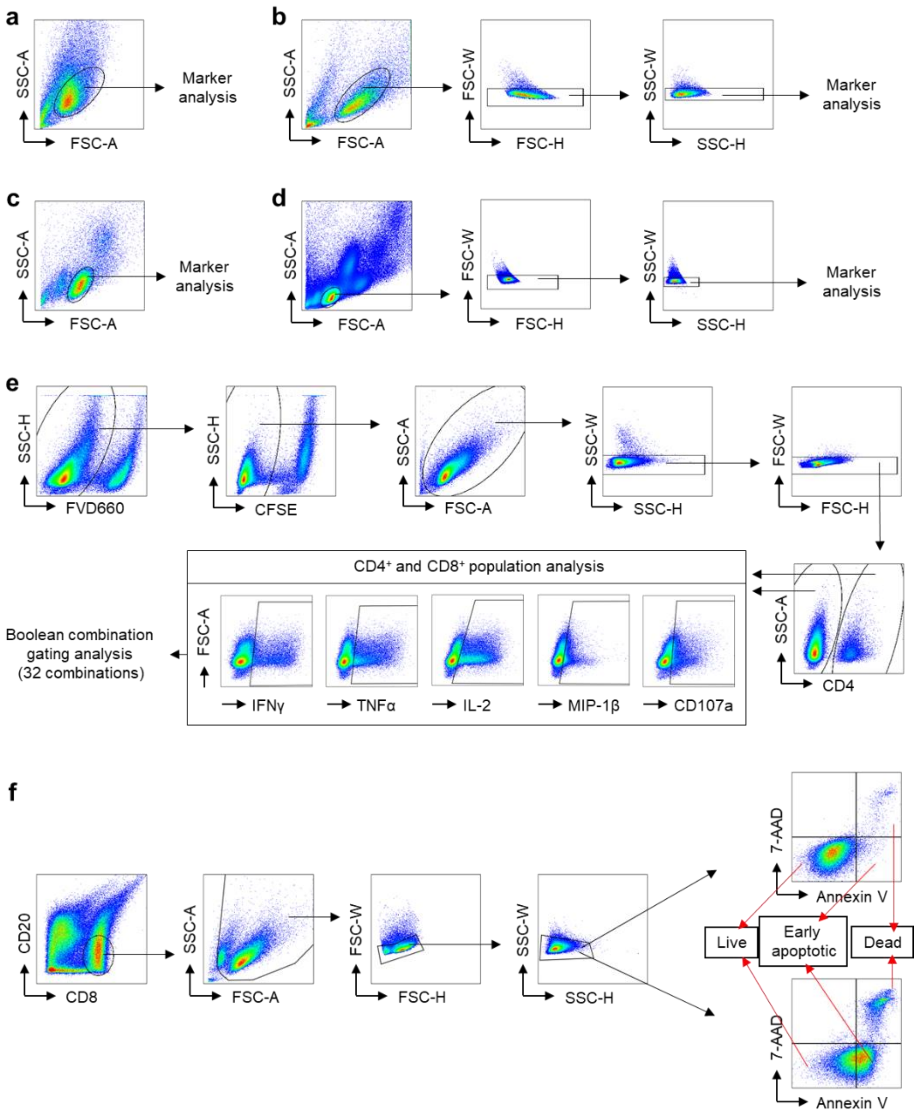

Supplementary Figure 2. Gating strategy. (a) To analyze cell lines using FACSCalibur (**Figure 1b,c and 3a**), viable cell population was gated and analyzed. **(b)** To analyze cell lines using FACSVerse (**Figure 4b and 5a,b**), singlet population in viable cells was gated and analyzed. **(c)** To analyze peripheral blood mononuclear cells using FACSCalibur (**Figure 3b**), viable cell population was gated and analyzed. **(d)** To analyze T cells using FACSVerse (**Figure 5c**), singlet population in viable cells was gated and analyzed. **(e)** Boolean combination gating for the polyfunctionality analysis (**Figure 6a,b**). Viable cell population (fixable viability dye eFluor 660-negative; $FVD660^-$) was gated and further separated from carboxyfluorescein succinimidyl ester (CFSE)-labeled LCLs. Within singlet population, $CD4^+$ or $CD4^-$ (i.e. $CD8^+$) cells were analyzed for each marker expression. The frequency of cell population with multiple marker expression was calculated by Boolean combination gating analysis. **(f)** Gating for the evaluation of apoptotic/dead cell frequency (**Figure 6g,h**). $CD8^+$ CAR-T population was gated by excluding $CD20^{high}$ LCLs. FSC-A/SSC-A gating was performed to exclude debris. Following singlet gating (FSC-H/FSC-W and SSC-H/SSC-W), the frequency of live (Annexin V^-/7-AAD^-), early apoptotic (Annexin V^+/7-AAD^-), and dead (Annexin V^+/7-AAD^+) cells among population was determined.

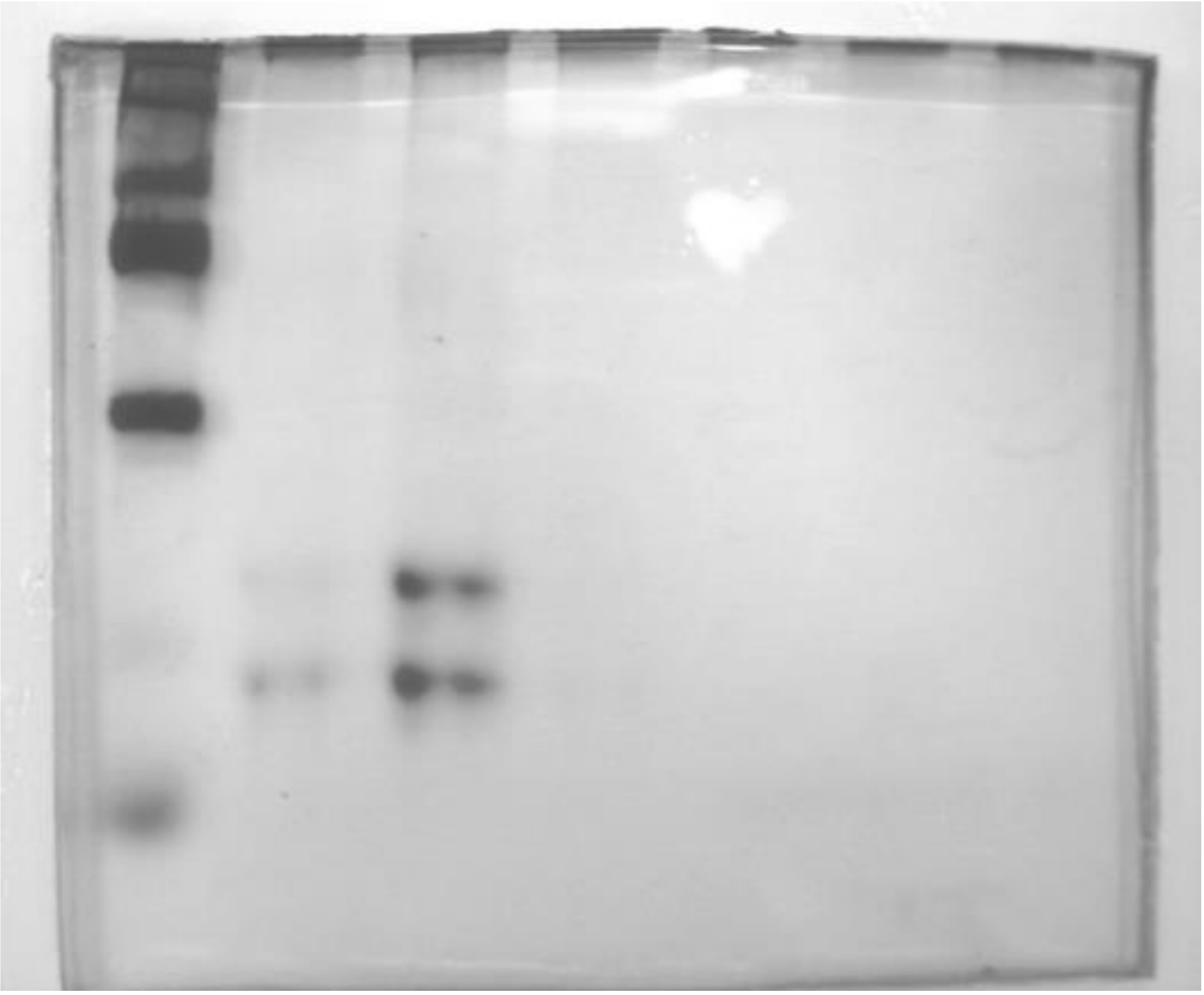

Supplementary Figure 3. Original untrimmed image of Figure 2b
